# A Review on Antidiabetic Activity of *Centaurea* spp.: A New Approach for Developing Herbal Remedies

**DOI:** 10.1155/2021/5587938

**Published:** 2021-07-05

**Authors:** Samaneh Fattaheian-Dehkordi, Reza Hojjatifard, Mina Saeedi, Mahnaz Khanavi

**Affiliations:** ^1^Department of Pharmacognosy, Faculty of Pharmacy, Tehran University of Medical Sciences, Tehran, Iran; ^2^Medicinal Plants Research Center, Faculty of Pharmacy, Tehran University of Medical Sciences, Tehran, Iran; ^3^Persian Medicine and Pharmacy Research Center, Tehran University of Medical Sciences, Tehran, Iran; ^4^Endocrinology and Metabolism Research Center, Endocrinology and Metabolism Clinical Sciences Institute, Tehran University of Medical Sciences, Tehran, Iran; ^5^Faculty of Land and Food Systems, University of British Columbia, Vancouver, Canada

## Abstract

**Objective:**

Diabetes mellitus (DM) is a long-life metabolic disorder, characterized by high blood glucose levels. The hyperglycemic condition generally leads to irreversible nerve injury and vascular damage. Among different types of diabetes, type 2 is more common and has spread all over the world. Although various therapeutic approaches have been developed to control type 2 DM, regulating blood glucose levels has still remained a controversial challenge for patients. Also, most prescription drugs cause different side effects, such as gastrointestinal disorders. Thus, developing novel and efficient antidiabetic agents possessing fewer adverse effects is in high demand.

**Method:**

The literature was comprehensively surveyed *via* search engines such as Google Scholar, PubMed, and Scopus using appropriate keywords.

**Results:**

Medicinal plants, both extracts and isolated active components, have played a significant role in controlling the blood glucose levels. Good-to-excellent results documented in the literature have made them a precious origin for developing and designing drugs and supplements against DM. *Centaurea* spp. have been traditionally used for controlling high blood glucose levels. Also, the antidiabetic properties of different species of *Centaurea* have been confirmed in recent studies through *in vitro* assays as well as *in vivo* experiments.

**Conclusion:**

Potent results encouraged us to review their efficacy to open a new horizon for development of herbal antidiabetic agents.

## 1. Introduction

Diabetes mellitus (DM) is a chronic metabolic disease which is described by hyperglycemia and high blood sugar levels in postprandial and fasting state. It is characterized by defects in insulin secretion, insulin action, or both of them [1]. The total number of diabetic patients in the world has been anticipated to rise from 171 million in 2000 to 366 million in 2030 [2]. Considering the long-term side effects of DM, it has become one of the major causes of morbidity in the world [3]. There are different types of diabetes based on its pathogenesis, including insulin-dependent (type I), noninsulin-dependent (type II), and gestational. Type 2 DM is more common than the other types in which the body's insulin receptors become resistant to the normal insulin effects. Then, *β* cells of the pancreas respond to the high blood glucose levels by producing more insulin to manage the situation. However, the insulin overproduction makes *β* cells wear themselves out [4, 5].

Patients with DM may experience some complications such as retinopathy, neuropathy, nephropathy, cataracts, peripheral vascular insufficiencies, and damaged nerves resulting from chronic hyperglycemia [5–7]. High blood glucose levels in type 2 DM can be controlled by using insulin or oral antidiabetic drugs [8]. Different pathways and mechanisms are considered for preventing the progression of the disease. They may include inhibition of intestinal *α*-glucosidase and *α*-amylase, inhibition of aldose reductase, insulin synthesis and secretion, inhibition of lens aldose reductase, oxidative stress protection, inhibition of formation of advanced glycation end products, lowering plasma glucose levels, altering enzyme activity of hexokinases and glucose-6‐phosphate, inhibition of postprandial hyperglycemia, stimulation of GLUT‐4, decreasing activity of G6P, and reducing the level of skeletal hexokinases [5].

One of the most popular approaches to the management of blood glucose levels is the inhibition of key enzymes [9]. *α*-Glucosidase and *α*-amylase are two carbohydrate digestive enzymes which can cause elevated postprandial hyperglycemia (PPHG); thus, their inhibition plays a significant role in controlling PPHG in patients with type 2 DM. Inhibition of *α*-glucosidase leads to the reduction of disaccharide hydrolysis, and inhibition of *α*-amylase disrupts the breakdown of starch to simple sugars. Some of these compounds are clinically used, and the results have shown significant reduction of blood glucose levels in patients [10, 11]. The most important side effect related to the approved Food and Drug Administration (FDA) antitype 2 DM drugs, including voglibose, acarbose, miglitol, sulphonylureas, and thiazolidine, is gastrointestinal problems such as swelling, abdominal distraction, diarrhea, and meteorism, which need more attention. Thus, investigation of different therapeutic agents with lower side effects is in high demand. Accordingly, herbal remedies have absorbed lots of attention [12–14] and different medicinal plants such as *Abelmoschus moschatus*, *Alangium salvifolium*, *Azadirachta indica*, *Bidens pilosa*, *Boerhaavia diffusa*, *Capsicum frutescens*, *Cassia alata*, *Eclipta alba*, *Embellica officinalis*, *Ficus carica*, *Gentiana olivier*, *Glycyrrhiza glabra*, *Gymnema sylvestre*, *Hordeum vulgare*, *Ipomoea aquatic*, *Juniperus communis*, *Mangifera indica*, *Momordica charantia*, *Ocimum sanctum*, *Punica granatum*, and *Zingiber officinale* have demonstrated enzyme inhibitory activity possessing desirable effects on diabetes and hyperglycemia [15–33]. Furthermore, various phytochemicals such as alkaloids, sesquiterpene and saponins, polysaccharides, flavonoids, dietary fibers, ferulic acid, tannins, limonene, and oleuropeoside have been studied for their inhibitory activity toward enzymes involved in the one set and progression of type 2 DM, which deserve to be considered for the development and production of herbal anti-DM supplements [5, 24, 34–43].

The genus *Centaurea* (family Asteraceae, tribe Cardueae, subtribe Centaureinae) compromises approximately 600 species worldwide, from Asia, Europe, and tropical Africa to North America [44]. *Centaurea* spp. have long been used in traditional medicine to cure various ailments such as diabetes, diarrhea, rheumatism, malaria, hemorrhoids, and neurological disorders. They have also been used in the treatment of inflammation, common cold, fever, cough, and ophthalmic disorders and their liver strengthening, wound healing, and anti-itching effects have been important [45–50]. A wide range of secondary metabolites, including sesquiterpene lactones (SLs) [44, 51–53], flavonoids [45, 46, 54, 55], lignans, and alkaloids [44, 45, 55], have been isolated from different *Centaurea* spp. The genus *Centaurea* is known for possessing sesquiterpene lactones (SLs) [56, 57] and phenolic compounds [58]. Herein, focusing on the hypoglycemic activity of various species of *Centaurea* in both folk and modern medicine [59–66], we reviewed different reports on their antidiabetic potency to develop herbal drugs and supplements for controlling blood sugar.

## 2. Methods

The literature was completely searched *via* search engines such as Google Scholar, Pub Med, and Scopus using keywords, including DM, *Centaurea*, hyperglycemia, medicinal plants, antidiabetic plants, *α*-glucosidase, *α*-amylase, high blood glucose levels, enzyme inhibition, plant-based diets, folk medicine, and treatment. All results were extracted and analyzed in a comprehensive manner.

## 3. Results

**Figure 1:**
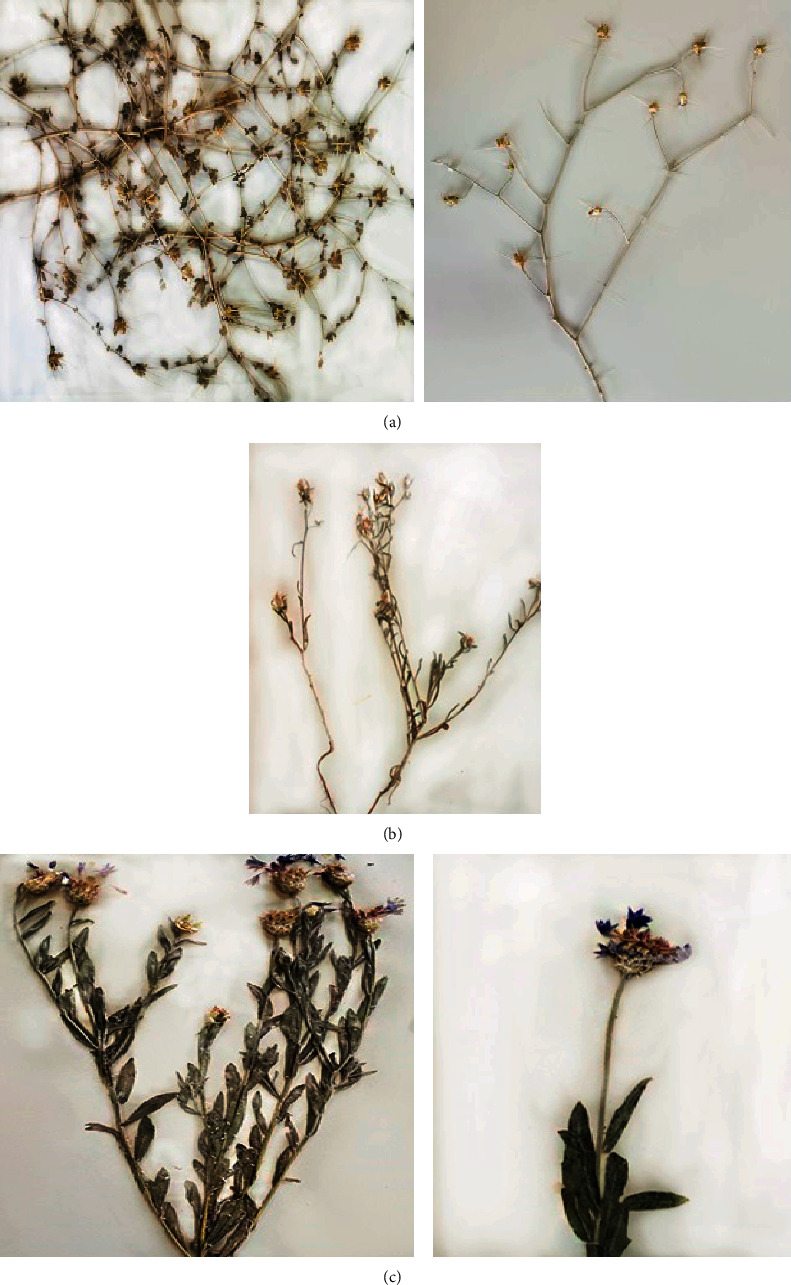
Some *Centaurea* species deposited in the herbarium of the Faculty Of Pharmacy, Tehran University of Medical Sciences. (a) *Centaurea bruguierana*. (b) *Centaurea patula*. (c) *Centaurea depressa*.

Antidiabetic activity of *Centaurea* spp. (Figure 1) has been usually investigated through the *in vitro* inhibition of *α*-glucosidase and *α*-amylase as well as *in vivo* studies on rats and mice (Table 1). However, no clinical trials have been conducted. *α*-Glucosidase and *α*-amylase are clinically responsible for glucose disorders in patients with type 2 DM. Reported results have been summarized in Table 1.

### 3.1. *In Vitro* Assays

#### 3.1.1. *Centaurea bornmuelleri*

*In vitro α*-amylase and *α*-glucosidase, as well as antioxidant activities of *Centaurea bornmuelleri*, have been reported in the literature. Among methanolic, aqueous, and ethyl acetate extracts of aerial parts of *C. bornmuelleri*, the ethyl acetate extract was found to be more potent than the others toward *α*-amylase and *α*-glucosidase [67] (Table 1). Other studies confirmed the antibacterial and antioxidant activity of the methanolic extract of the plant [80]. Also, it could inhibit the growth of colon cancer cells under *in vitro* conditions [81].

#### 3.1.2. *Centaurea calcitrapa*

*Centaurea calcitrapa* has been used in folk medicine for the treatment of ophthalmic and skin diseases, common fever, jaundice, and digestive disorders [82–84]. In an *in vitro* study, the antidiabetic activity of methanolic extract of aerial parts of the plant was investigated. It could inhibit *α*-glucosidase with IC_50_ value of 4.38 ± 0.31 mg/ml comparing with acarbose (IC_50_ = 1.41 ± 0.07 mg/ml) [68] (Table 1). It is worth mentioning that the extract has also shown antibacterial activity against *Bacillus*, *Pseudomonas*, *Staphylococcus*, *Streptococcus*, *Salmonella*, *Enterobacter*, *Enterococcus, Acinetobacter*, and *Escherichia* genera [85–87]. Furthermore, *C. calcitrapa* has depicted significant antioxidant activity through *β*-carotene/linoleic acid bleaching assay. *In vivo* antioxidant assay in mice at the doses of 50 and 100 mg/kg/day within 21 days afforded a protective effect against erythrocytes hemolysis [88].

#### 3.1.3. *Centaurea centaurium*

*In vitro α*-amylase inhibitory activity of methanolic, aqueous, polyphenol, and *n*-hexane extracts of *Centaurea centaurium* was assayed by Conforti et al. [69]. The *n*-hexane extract was the most potent extract with an IC_50_value of 158 *μ*g/ml. However, aqueous and polyphenol extracts were inactive, and the methanolic extract was found to be weak with an inhibition percent of 32.51 ± 0.34% at the concentration of 1000 *μ*g/ml.

#### 3.1.4. *Centaurea depressa, Centaurea drabifolia, Centaurea kotschyi, Centaurea patula, Centaurea pulchella, Centaurea tchihacheffii, Centaurea triumfettii,* and *Centaurea urvillei*

The chloroform and ethyl acetate extracts of aerial parts of eight *Centaurea* spp. including *C. depressa*, *C. drabifolia*, *C. kotschyi*, *C. patula*, *C. pulchella*, *C. tchihacheffii*, *C. triumfettii*, and *C. urvillei* were investigated for their *α*-glucosidase and *α*-amylase inhibitory activity by Zengin et al. All *Centaurea* spp. extracts were able to inhibit both enzymes at the concentration of 2 mg/mL (Table 1) and compared with acarbose, inducing inhibitory activity toward *α*-amylase and *α*-glucosidase with inhibition percent of 50.51% and 44.16% at 1 mg/ml. The chloroform extract of *C. pulchella* and *C. depressa* and the ethyl acetate extract of *C. urvillei* showed the most potent *α*-amylase inhibitory effects with inhibition percent of 59.54%, 43.97%, and 43.20%, respectively. The antiglucosidase effect was reported in the following order: ethyl acetate extract of *C. triumfettii* (69.88%) > ethyl acetate extract of *C. urvillei* (67.66%) > chloroform extract of *C. pulchella* (60.31%) [70].

It should be mentioned that antioxidant, antibacterial, antinociceptive, antipyretic, and anticholinesterase activities of these species were also proven [14, 70, 89–93].

#### 3.1.5. *Centaurea fenzlii*

The methanolic extract of *Centaurea fenzlii* has shown *α*-glucosidase and *α*-amylase inhibitory activity as 0.331 mmol ACAE/g dry weight and 0.354 mmol ACAE/g dry weight, respectively [71]. The plant has also shown antioxidant, antityrosinase, and anticholinesterase activities, as well as cytotoxicity against colon and MCF-7 breast cancer cell lines [71, 94, 95].

#### 3.1.6. *Centaurea hypoleuca*

Ethanolic, methanolic, and ethyl acetate extracts of aerial parts (flower and stem) of *Centaurea hypoleuca* have depicted *in vitro* inhibitory activity toward *α*-glucosidase and *α*-amylase. It should be noted that the ethyl acetate extract of the plant flowers resulted in higher activity than that of the stem as well as other extracts (Table 1) [72]. Also, all extracts demonstrated moderate-to-good antioxidant, antimicrobial, and anticholinesterase activities [72].

#### 3.1.7. *Centaurea karduchorum*

The dried powder of *Centaurea karduchorum* has been traditionally used for wound healing [96]. Also, tea prepared from aerial parts of the plant was found to be helpful for the treatment of diabetes, which was investigated and proven in recent studies. Among ethanolic extracts obtained from roots, stems, leaves, and flowers of the plant (Table 1), the leaves extract showed the best inhibitory activity against *α*-glucosidase (IC_50_ = 0.63 ± 0.00 mg/ml); however, it could not efficiently inhibit the *α*-amylase (IC_50_ = 14.63 ± 0.67 mg/ml) [73].

Comparing *α*-glucosidase inhibitory activity of *C. karduchorum* with that of cinnamon which is known for its antidiabetic activity revealed potent efficacy of *C. karduchorum* since the activity of various extracts of cinnamon was calculated in the range of IC_50_ = 0.42–4.0 mg/ml [73, 97].

#### 3.1.8. *Centaurea papposa*

*In vitro α*-glucosidase inhibitory activity of *n*-butanol, dichloromethane, and ethyl acetate extracts of *Centaurea papposa* was studied by Mawahib et al. Among them, dichloromethane extract displayed a greater inhibitory activity (IC_50_ = 227.6 ± 4.4 *μ*g/ml) comparing with acarbose (275.4 ± 1.6 *μ*g/ml). The ethyl acetate extract exhibited weak anti-*α*-glucosidase activity (IC_50_ = 791.9 ± 1.8 *μ*g/mL), and the *n*-butanol extract, however, was inactive [8].

#### 3.1.9. *Centaurea saligna*

*Centaurea saligna* has been traditionally used as a wound healing agent, astringent, and tonic. Moreover, its choleretic, diuretic, antibacterial, antirheumatic, and antipyretic activities have been reported [49, 74, 98]. The plant also has demonstrated anticholinesterase, antityrosinase, antiradical, antimicrobial, and antiproliferative properties on LNCaP, HCT-116, and MCF-7 cancer cell lines [74, 99, 100].

Methanolic, aqueous, and ethyl acetate extracts of *C. saligna* leaves were studied against *α*-glucosidase (3.32–23.80 mmol ACAE/g extract) and *α*-amylase (0.16–0.80 mmol ACAE/g extract) by Zengin et al. Among them, the ethyl acetate extract showed the most potent anti-*α*-glucosidase activity (23.80 mmol ACAE/g extract). It is clear that those extracts exhibited weak inhibitory activity toward *α*-amylase [74].

#### 3.1.10. *Centaurea triumfettii*

Leaves of *Centaurea triumfettii* have been traditionally used as foodstuff [92, 101]. Biological activities of methanolic, ethanolic, and ethyl acetate extracts of stems and flowers of *Centaurea triumfettii* have been reported by Acet [14]. The ethyl acetate extract of the stems showed potent inhibitory effects on *α*-amylase (165.47 ± 0.72 mmol ACAE/g extract) and *α*-glucosidase (4.13 ± 0.04 mmol ACAE/g extract). The plant has also shown the antioxidant capacity and antibacterial activity [14, 91, 102].

### 3.2. *In Vivo* Assay

#### 3.2.1. *Centaurea alexanderina*

*Centaurea alexanderina* has shown different biological activities such as anti-inflammatory, analgesic, hepatoprotective, and antibacterial (against *Pseudomonas aeruginosa*) effects and cytotoxicity on A-495 lung cancer cells [75, 103].

Antidiabetic properties of the 80% methanolic extract of leaves of *C. alexanderina* at the doses of 300 and 600 mg/kg have been studied under *in vivo* conditions in normoglycemic as well as streptozotocin- (STZ-) induced diabetic rats. Those results were compared with glibenclamide (50 mg/kg) as the standard drug. Administration of the extract at the dose of 600 mg/kg led to a remarkable reduction of the elevated blood glucose by 9.4% and 10.5% after 1 and 2 h, respectively. However, using the dose of 300 mg/kg decreased the related item to 2.8% after 2.5 h. Using 300 and 600 mg/kg of extracts daily within two months in the STZ-induced diabetic model led to the reduction of plasma glucose levels by 2.7% and 4.9%, respectively. However, the reduction of test days to 30 days affected the efficacy of extract, and the corresponding levels reduced to 1.1% and 3.8%, respectively [75].

#### 3.2.2. *Centaurea aspera*

Aqueous extracts of *Centaurea aspera* flowers were investigated for their hypoglycemic activity in normal and alloxan-diabetic rats. It exhibited an important hypoglycemic effect by oral route and chronic administration in diabetic rats comparing with glibenclamide. It should be mentioned that the extract obtained by exhaustion with hot water showed an acute hypoglycemic activity in normal animals [76].

#### 3.2.3. *Centaurea bruguierana*

Hypoglycemic activity of different extracts of *Centaurea bruguierana* and the mechanism of action was investigated in STZ-alloxan-diabetic rats by Khanavi et al. The aqueous and dichloromethane extracts at the dose of 400 mg/kg and the ethyl acetate and methanol extracts at the dose of 200 mg/kg, obtained from aerial fruiting parts of the plant, were investigated. The ethyl acetate extract afforded the best activity to reduce the blood glucose levels up to 50.0%, while methanol, dichloromethane, and aqueous extracts reduced that up to 45.7%, 41.7%, and 29.5%, respectively. Glibenclamide showed a 34.5% reduction. The best result from reduction of phosphoenolpyruvate carboxykinase (PEPCK) activity (84.0%) and increasing hepatic glycogen phosphorylase (GP) activity (134.5%) points of view was related to the aqueous extract comparing with those of glibenclamide (62.5% and 133.0%), respectively. *C. bruguierana* depicted no effect on blood insulin, but it was able to reduce blood glucose by stimulation of hepatic glycogenolysis and inhibition of gluconeogenesis [77, 104].

#### 3.2.4. *Centaurea corubionensis*

Chuclá et al. studied the effect of aqueous and ethanolic extracts of leaves and flowers of *Centaurea corubionensis* on normoglycemic rats, circulating insulin levels in anesthetized rats, glucose-induced hyperglycemic rats, and alloxan-diabetic rats at different doses of 2.5, 5, and 10 g/kg [78]. Consumption of aqueous extracts of leaves and flowers at the dose of 5 g/kg led to the reduction of blood glucose levels by 19 and 16%, respectively. Also, 6 h after administration of aqueous extract of leaves (5 g/kg), the serum glucose and insulin levels were reported to be 97.2 (mg%) and 10.2 (*μ*U/ml) comparing with tolbutamide (75 mg/kg) with those values of 84.4 (mg%) and 9.2 (*μ*U/ml), respectively. Moreover, aqueous extract of flowers (50 mg/ml) could increase insulin release from isolated islets of Langerhans to 36 *μ*U/ml. However, no effect was observed on alloxan-diabetic animals, and it may be associated with severe damage of the pancreas by the alloxan. Hypoglycemic properties of *C. corubionensis* can be achieved by the undamaged pancreas *via* raising serum circulating insulin.

#### 3.2.5. *Centaurea horrida*

Raafat et al. investigated the antidiabetic effect of the methanolic extract of *Centaurea horrida* herb and roots in alloxan-induced diabetic mice comparing with glibenclamide. All results were generally obtained more significantly than those of glibenclamide. The plant has been traditionally used to lower blood glucose levels [79]. It was found that administration of the extract at dose of 100 mg/kg led to the reduction of blood glucose levels from 219.33 to 106.56 mg/dL. Investigation of the subacute effect of the extract exhibited the reduction of blood glucose levels from 121.84 mg/dL on 1th day to 105.42 mg/dL on the 8th day at the same dose. The subacute effect of the extract on body weight in alloxan-induced diabetic mice also revealed that using the extract at different doses of 5, 25, 50, and 100 mg/kg did not lead to a significant overweight in mice which was comparable to the positive control. *In vivo* assessment of the antioxidant activity of the extract demonstrated that treated mice with doses of 25, 50, and 100 mg/kg had no remarkable increase in serum catalase activity. However, it was clear that long-term treatment of diabetes with all doses, particularly with a high dose of extract, induced a reversed effect on catalase activity, which may be associated with reduced oxidative stress. It is worth mentioning that using the extract significantly improved peripheral nerves function of diabetic mice *via* hot plate and tail flick tests. This is an important result as uncontrolled high blood glucose levels can damage peripheral nerves causing diabetic neuropathy [79, 105, 106]. It has been suggested that hypoglycemic effect of the plant is achieved by the inhibition of the endogenous glucose production or inhibition of intestinal glucose absorption and controlling dietary glucose uptake in the small intestinal tract. It is believed that the mechanism is independent of insulin secretion [79].

The elastase and tyrosinase inhibitory effects of *C. horrida* have also been reported [107].

## 4. Discussion

Herbal medicine has occupied a particular position in healing purposes, and their use has grown significantly over recent years. In this respect, there are a wide range of reports on the antidiabetic activity of medicinal plants [108], which can be fully considered for the development of efficient drugs and supplements.

### 4.1. Toxicity

It should not be forgotten that all natural remedies are not essentially safe, and all herbal medicine users should be aware of the risks that they carry [93, 109]. To reach this goal, the toxicity of plants should be investigated for better knowing the range of safety. According to the literature, there are no enough data on the toxicity of reported *Centaurea* spp. in this paper, and most plant toxicity tests should be conducted.

Orally administration of 80% methanolic extract of *C. alexanderina* by different groups of mice (*n* = 10) in the dose range of 50–3000 mg/kg resulted in no fatality and the LD_50_ value was assumed to be greater than 3000 mg/kg [75].

LD_50_ value for the methanolic extract of *C. urvillei* was calculated as 115.5 × 10^−2^ using the brine shrimp lethality bioassay [110]; likewise, the LC_50_ values for methanolic and diethyl ether extracts of *C. triumfettii* were obtained as 266.5 and 166.6 *μ*g/ml, respectively [111].

Cytotoxicity of petroleum ether, chloroform, ethyl acetate, *n*-butanol, and remaining methanolic fractions of the methanolic extract of *C. bruguierana* depicted that petroleum ether and remaining methanolic fractions were nontoxic toward NIH-3T3 cells (Swiss embryo fibroblast) [112]. However, in a study reported by Nasr et al. [113], chloroform, ethyl acetate, *n*-butanol, and methanol fractions of the plant showed toxicity on HUVEC cells (a noncancerous cell line).

As reported by Erol-Dayi et al. [114], evaluation of cytotoxicity of methanolic and aqueous extracts of *C. calcitrapa*, *C. ptosimopappa*, and *C. spicata* indicated the lack of toxicity of aqueous extract of *C. ptosimopappa* and *C. spicata* on Hela (human cervix adenocarcinoma) and Vero (normal African green monkey kidney) cells (IC_50_ > 1000 *μ*g/ml). Those methanolic extracts were found to be more toxic (IC_50_ > 200 *μ*g/ml) on the same cells. The aqueous extract of *C. calcitrapa* showed moderate toxicity on both cells (IC_50_ > 400 *μ*g/ml), whereas the methanolic extract demonstrated an inhibitory effect with IC_50_ < 100 *μ*g/ml on Hela and Vero cells (92.5 and 91.7 *μ*g/mL, respectively). It indicated that the methanolic extract of *calcitrapa* needs more attention from the toxicity point of view.

According to the results reported by Conforti et al. [69], based on the brine-shrimp toxicity test on the roots of *C. centaurium*, the LC_50_value was calculated as 44.05 mg/ml for the methanolic extract, while LC_50_values for the polyphenolic, lipophilic, and water fractions were found to be 157.44, 25.98, and 152.81 mg/ml, respectively.

### 4.2. Constituents Isolated from *Centaurea* spp. and Their Antidiabetic Activity Mechanism of Action (MOA)

**Figure 2:**
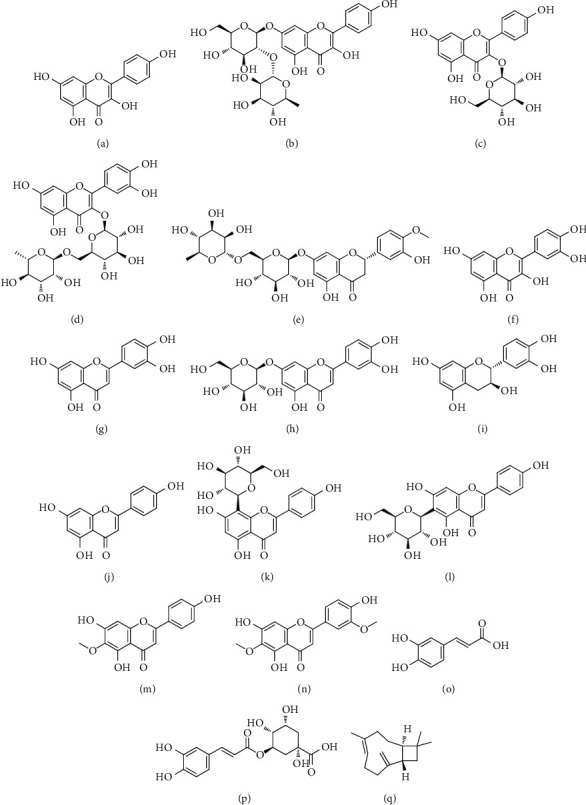
The chemical structure of constituents isolated from *Centaurea* spp., responsible for antidiabetic activity. (a) Kaempferol. (b) Kaempferol 3-*O*-rutinoside. (c) Astragalin (kaempferol-3-glucoside). (d) Rutin. (e) Hesperidin. (f) Quercetin. (g) Luteolin. (h) Cynaroside (luteolin-7-*O*-glucoside). (i) Catechin. (j) Apigenin. (k) Vitexin. (l) Isovitexin. (m) Hispidulin. (n) Jaceosidin. (o) Caffeic acid. (p) Cholorogenic acid. (q) *β*-Caryophyllene.

The antidiabetic activity of *Centaurea* spp. is definitely indebted to the presence of phytochemicals. Isolated constituents from discussed plants are listed in Table 2. In this respect, sesquiterpenes, flavonoids, and phenolic compounds have been generally reported in the literature (Figure 2).

#### 4.2.1. Sesquiterpene Lactones

Sesquiterpenoids have shown potent antidiabetic activity *via* various mechanisms such as inhibition of enzymes involved in hyperglycemia, protecting *β*-pancreatic cells, preventing oxidative and inflammatory damages associated with the disease, and improving insulin secretion. They can improve insulin sensitivity by regulating glucose transport and key proteins of the insulin signaling pathway. They have also exhibited lipid-lowering actions [158].

Sesquiterpene lactones have exhibited hypoglycemic effects in STZ-induced diabetic mice by improving the function of pancreatic islets, increasing glycolysis, and decreasing gluconeogenesis as well as antioxidant and hypolipidemic activities, which have been assessed by using *in vitro* assays. The mechanism of antidiabetic activity may involve an antioxidant effect, improving insulin sensitivity, and stimulation of pancreatic *β*-cells to secret insulin [159]. Sesquiterpene lactones have also shown *in vitro* inhibitory effects on *α*-glucosidase and *α*-amylase [160]. They can be used for the treatment of diabetes through the regulation of nuclear factor kappa-light-chain-enhancer of activated B cells (NF-*κ*B) and mitogen-activated protein kinase (MAPK) signaling pathway [158, 161]. They have also reduced the production of chemokines, such as MCP-1, TGF-*β*1, and FN, activate NF-*κ*B, and inhibited sugar-induced degradation of I*κ*B*α*, confirming the efficacy of sesquiterpene lactones as drug candidates for the treatment of diabetic nephropathy [158, 162].

*β*-Caryophyllene, as a sesquiterpene lactone derivative, has shown antihyperglycemic activity in STZ-induced diabetic rats. Oral administration of *β*-caryophyllene significantly decreased glucose and increased insulin levels. Moreover, reversing the glycoprotein levels in plasma and tissues of diabetic rats to near normal and decreasing proinflammatory cytokines detected using histological and immunohistochemical studies demonstrated the antioxidant capacity of this compound [163, 164]. It should be noted that chronic use of *β*-caryophyllene has also depicted good results in the prevention or reduction of diabetes-related neuropathy and depressive-like behavior in mice (assessed by marbles test) [165].

#### 4.2.2. Flavonoids

Flavonoids are one of the major components of *Centaurea* spp. Four flavonoids including scutellarein, nepetin, apigenin, and hispidulin were evaluated for their *α*-glucosidase inhibitory effects comparing with acarbose and the order of the activity was obtained as scutellarein > nepetin > apigenin > hispidulin > acarbose. Also, the synergistic effects from the combination of each flavonoid with acarbose at different concentrations were observed. It was perceived that the best synergistic effect was related to the combined apigenin-acarbose which acted as a noncompetitive inhibitor [166].

The antihyperglycemic effect of apigenin may be related to the inhibition of *α*-glucosidase, preventing oxidative stress conditions, decreasing insulin resistance, decreasing hepatic gluconeogenic enzymes activity, and increasing serum insulin levels [167–169]. Apigenin can enhance the metabolism of glucose *via* suppression of the activities of gluconeogenic enzymes and aldose reductase. It also prevents diabetic complications such as cataracts, retinopathy, and neuropathy due to the intracellular sorbitol accumulation. Glucose is converted to sorbitol in the polyol pathway, catalyzed by aldose reductase [170].

Vitexin and isovitexin are two apigenin isomers, and their *α*-amylase inhibitory effects and antioxidant potentials have been investigated *viain vitro* assays. Vitexin and isovitexin exhibited significant anti-*α*-amylase activity with IC_50_values of 4.6 and 13.8 *μ*M, respectively. Also, antioxidant activity was assayed through DPPH free radical scavenging assay, which showed IC_50_ values of 92.5 and 115.4 *μ*M, respectively [171]. Vitexin also depicted inhibitory effect on *α*-glucosidase (IC_50_ = 52.805 *μ*M) which was comparable with that of acarbose (IC_50_ = 375 *μ*M) [172]. In addition, computer-aided studies of vitexin-amylase, isovitexin-amylase, and vitexin-glucosidase complexes in the active site of related enzymes confirmed the construction of desired interactions with amino acid residues [171, 172]. Another *in vitro* study using cell culture revealed that vitexin protected pancreatic *β*-cells from high-glucose-induced damage, inhibited islet *β*-cell apoptosis, and improved insulin release and sensitivity. The underlying mechanism may increase the expression of transcription factor Nrf2, resulting in increased intracellular antioxidant molecules, and suppress the inflammatory signaling pathway. Besides, vitexin enhances insulin production by activating insulin signaling *via* the activation of phosphorylation of IR, IRS-1, and IRS-2 [173].

Hispidulin is another important flavonoid compound inducing antidiabetic activity. Oral administration of hispidulin to STZ-induced hyperglycemia mice effectively mitigated postprandial and fasting hyperglycemia and glucose tolerance, which was associated with a dual mechanism, promoting *β*-cell function and suppressing hepatic glucose production [174].

Kaempferol has also depicted remarkable *α*-glucosidase and *α*-amylase inhibitory activity [175, 176]. Oral administration of kaempferol significantly improved blood glucose control in obese mice, which was associated with suppressing hepatic gluconeogenesis and improving insulin sensitivity and secretion [177, 178]. It was found that kaempferol-3-*O*-rutinoside was also a potent inhibitor of *α*-glucosidase, being over 8 times more active than the reference drug, acarbose, under *in vitro* conditions [179].

Astragalin has shown hypoglycemic activity on Wistar rats (10 mg/kg) and improved insulin secretion in the glucose tolerance test. Investigation of isolated pancreatic cells treated with astragalin (100 *μ*M) led to Ca^2+^ influx stimulation *via* a mechanism involving ATP-dependent potassium channels, L-type voltage-dependent calcium channels, the sarco/endoplasmic reticulum calcium transport ATPase (SERCA), and PKC and PKA (protein kinase) [180].

Rutin is also an important flavonoid possessing antihyperglycemic effects *via* various mechanisms, including decrease of carbohydrates absorption from the small intestine, inhibition of tissue gluconeogenesis, increase of tissue glucose uptake, stimulation of insulin secretion from *β*-cells, and protecting Langerhans islet against degeneration. Rutin also decreases the formation of sorbitol, reactive oxygen species, advanced glycation end-product precursors, and inflammatory cytokines [181].

Luteolin and luteolin 7-*O*-glucoside have shown good *α*-glucosidase inhibitory activity. However, luteolin was found to be more potent than acarbose by the inhibition of 36% at the concentration of 0.5 mg/ml. Although luteolin could inhibit *α*-amylase effectively (IC_50_ in the range of 50 to 500 *μ*g/ml), it was less potent than acarbose [182].

Jaceosidin is another flavonoid compound, and its antihyperglycemic capacity has been assessed through various *in vivo* studies. The results showed that jaceosidin supplementation significantly lowered fasting blood glucose levels and reduced insulin resistance. As it was also found that jaceosidin supplementation increased antioxidant capacity by enhancement of catalase and GSH-px activities, a relevant relationship between antioxidant and antihyperglycemic effects of jaceosidin can be concluded. Jaceosidin could improve endoplasmic reticulum stress and attenuate insulin resistance *via* SERCA2b (sarco/endoplasmic reticulum Ca^2+^-ATPase 2b) upregulation in mice skeletal muscles [183, 184].

Hesperidin has shown antidiabetic activity. It has inhibited obesity, hyperglycemia, and hyperlipidemia, and decreased insulin resistance. These effects might be closely related to the activation of AMPK, which regulate the insulin signaling pathway and lipid metabolism [185]. Hesperidin ameliorates pancreatic *β*-cell dysfunction and apoptosis in a streptozotocin-induced diabetic rat model [186].

The antidiabetic activity of quercetin is also important. It has reduced fasting and postprandial hyperglycemia in an animal model of DM [187]. An *in vivo* study revealed the hypoglycemic effects of quercetin, but no changes were observed in the activity of lipogenic enzymes and lipoprotein lipase. It can be concluded that the antidiabetic activity of quercetin is comparable with that of antiobesity activity [188]. There are different reports on the *α*-glucosidase inhibitory effect of quercetin, which describe its multilateral antidiabetic activity [187, 189, 190].

Oral administration of catechin to STZ-induced diabetic rats resulted in a potential agonist characteristic that is capable of activating the insulin receptors and producing a glucose tolerance pattern. The hypoglycemic effect of catechin is associated with its insulin mimetic activity [191]. It has been indicated that catechin significantly decreased the different lipid parameters, hepatic, and renal function enzyme levels along with HbA1c levels in diabetic rats while remarkably increased the high-density lipoprotein (HDL) levels with values comparable with the glibenclamide. Also, *α*-glucosidase and *α*-amylase inhibitory activity of catechin have been reported with inhibition percent of 80% and 79%, respectively [192].

#### 4.2.3. Phenolic Compounds

Phenolic compounds have shown versatile and attractive antidiabetic activity. Caffeic acid, a known phenolic acid compound, could protect mice pancreatic islets from oxidative stress induced by multiwalled carbon nanotubes (MWCNTs) [193]. Investigation of the effect of caffeic acid and cinnamic acid on glucose uptake in TNF-R-induced insulin-resistant hepatocytes showed that they may eliminate insulin resistance by improving insulin signaling and enhancing glucose uptake in insulin-resistant cells, which described their antihyperglycemic potential [194]. In another report, glucose uptake into the isolated adipocytes was raised by caffeic acid. The increase of glucose utilization by caffeic acid seems to be responsible for lowering plasma glucose [195].

Chlorogenic acid could also reduce fasting blood glucose levels [196–198]. It has shown an inhibitory effect on *α*-amylase as potent as acarbose; however, its *α*-glucosidase inhibitory activity was far weaker than that of acarbose [199, 200].

The effect of phenolic compounds, particularly in the management of type 2 diabetes, has attracted lots of attention [201]. They are characterized by the presence of hydroxyl group(s) on the aryl moiety and endorsed by their antioxidant activity due to high potency of hydroxyl groups as hydrogen donors [202]. As it has been accepted that the formation of reactive oxygen species (ROS) is associated with hyperglycemia [203], using antioxidants is preferred to treat and reduce the complications of DM. Also, it has been proven that consuming a diet low in fat and rich in antioxidants may reduce the risk of obesity and insulin resistance [204–207].

Phenolic compounds comprise a wide range of phenolic acids and flavonoids. Flavonoids in turn contain anthocyanin pigments, flavonols, flavones, flavanols, and isoflavones. Polymerization of flavanols leads to the formation of tannins in which the esterification of phenolic groups affords cyclic chromenones such as ellagic acid. However, condensed tannins known as proanthocyanidins, for example, catechin, epicatechin, and gallocatechin, are obtained from the condensation of flavanols [208].

*Centaurea* spp. have been frequently reported to possess anthocyanins [207, 209–211] and their biological activities such as antioxidant, antiallergic, anti-inflammatory, antiviral, antiproliferative, antimutagenic, antimicrobial, and anticarcinogenic activities. Also, different properties such as improvement of microcirculation, protection from cardiovascular damage and allergy, prevention of peripheral capillary fragility, prevention of diabetes, and vision improvement are fully considered in the literature [207, 212–222]. Also, the role of anthocyanins is well described for their effect on the prevention of diabetic cataracts [207, 218, 223]. The presence of apigenin in *Centaurea* spp. [224] has been confirmed, and its activity against thyroid neoplasms as well as anxiolytic, anti-inflammatory, and antinociceptive properties has been reported [225–227]. The presence of flavonoids in *C. bornmuelleri* is significant and might be responsible for the desired activity [67]. The phytochemical analysis of *C. calcitrapa* proved the presence of sterols, sesquiterpene lactones, and their closely related group of triterpenoids, bisabolenes, lignans, and flavonoids as the main secondary metabolites [124–130]. *C. hypoleuca* contains higher amounts of catechin and chlorogenic acid than the other phenolic compounds, which are known to be responsible for various biological activities such as antioxidant, neuroprotective, antidiabetic, hepatoprotective, and antiarthritic properties [72, 147–149]. High levels of apigenin (2472 *μ*g/g extract), known as a common dietary flavonoid, has absorbed attention in *C. saligna*. *In silico* study has confirmed the construction of H-bonding and pi-pi stacking interactions between apigenin and the *α*-glucosidase active site [74]. Chlorogenic acid has been identified as the main phenolic compound in *C. triumfettii* [14]. *C. karduchorum* is known to possess abundant amounts of phenolic compounds, mainly luteolin glycosides (glucoside and glucuronide) and chlorogenic acid [73]. Some studies confirmed the activity of luteolin and/or its glycosides against diabetes and neurodegenerative diseases through the reduction of glucose uptake, oxidative stress, and inflammation [151]. Chlorogenic acid has chemopreventive and hypoglycemic effects [150], and it is the main component of medicinal plants characterized by their antioxidant, anti-inflammatory, and enzyme inhibitory activities [150, 189, 228]. *C. bruguierana* possessed sesquiterpene lactones and flavonoids (kaempferol, rutin, and quercetin) [77, 104, 120]. Also, the plant has been documented for its antiplasmodial and antipeptic ulcer effects [77, 229, 230]. The antidiabetic property of *C. karduchorum* as a herbal tea is directly dependent on the high levels of bioactive phenolic derivatives profiting from synergistic interactions of those compounds [73]. The presence of terpenes has been confirmed through qualitative analysis in *C. papposa*, which may explain the favorite activity toward *α*-glucosidase [154]. High total phenolic and flavonoid contents of *C. pulchella* and *C. urvillei*, respectively, may explain their antidiabetic activity [70]. Phytochemical examination of aerial parts of *C. horrida* indicated the presence of pentacyclic triterpenes, sterol glucoside, quinic acid derivatives, phenolic acid derivatives, and flavonoids as well as horridin [143, 144].

As mentioned above, discussed species of *Centaurea* are known to possess a high content of phenolic compounds, which explains their antitype 2 DM activity.

Inhibition of *α*-glucosidase and *α*-amylase has been found to be a versatile tool for the treatment of type 2 diabetes [231, 232]. Apart from synthetic compounds [233–237], a wide spectrum of medicinal plants have been introduced to possess those enzymes inhibitory activity [238], and flavonoids have been well described in this field [239]. Amphiphilic property of phenolic moiety provides favorite interactions with enzymes *via* the construction of H-bonding and hydrophobic interactions with the polar groups of enzymes and hydrophobic amino acid residues, respectively.

An important point comes back to side effects related to *α*-amylase inhibitors. They include abdominal distention, flatulence, meteorism, and possibly diarrhea which are consequence of high activity of the enzyme. It seems that extreme inhibition of pancreatic *α*-amylase results in the abnormal bacterial fermentation of undigested carbohydrates in the colon [240–242]. In this respect, dual inhibitors such as *C. saligna* and *C. karduchorum* possessing weak inhibition of *α*-amylase and high inhibition of *α*-glucosidase are desirable for the treatment of type 2 DM.

Finally, the efficacy of *Centaurea* spp. under *in vivo* conditions has followed various mechanisms such as lowering blood glucose levels, stimulation of hepatic glycogenolysis, inhibition of gluconeogenesis, and insulin secretion and circulation.

## 5. Conclusion

In conclusion, the antidiabetic activity of some *Centaurea* spp., which has been studied for controlling hyperglycemia, was reviewed. The results obtained from *in vitro* and *in vivo* studies confirmed the efficacy of *Centaurea* spp. for the treatment of type 2 DM. *In vitro* assays generally focused on the *α*-glucosidase and *α*-amylase inhibitory activity, and the effectiveness of *C. bornmuelleri*, *C. calcitrapa*, *C. centaurium*, *C. drabifolia*, *C. depressa*, *C. fenzlii*, *C. hypoleuca*, *C. karduchorum, C. kotschyi*, *C. papposa, C. patula*, *C. pulchella*, *C. saligna*, *C. tchihacheffii*, *C. triumfettii*, and *C. urvillei* has been investigated. Among them, dichloromethane extract of *C. papposa* was found to be the most potent inhibitor of *α*-glucosidase, and the *n*-hexane extract of roots of *C. centaurium* showed the highest activity toward *α*-amylase (Table 1). *In vivo* studies of *C. alexanderina*, *C. aspera*, *C. bruguierana*, *C. corubionensis*, and *C. horrida* revealed that *C. horrida* and *C. bruguierana* were found to be more potent than glibenclamide and *C. corubionensis* was comparable with tolbutamide. These results demonstrated that *Centaurea* spp. deserve to be widely studied through clinical trials to prove their antidiabetic effects. Also, data related to the acute and chronic toxicity are in high demand to develop safe *Centaurea* spp.-based supplements and drugs against type 2 DM.

## Figures and Tables

**Table 1:** Antidiabetic activity of *Centaurea* spp.

Entry		*Centaurea* spp.	Action	Part	Extract	Activity^a^	Reference
1	*In vitro* studies	*C. bornmuelleri*	*α*-Glucosidase inhibition	Aerial parts	Ethyl acetate	33.12 ± 0.32 (mg ACAE^b^/g extract)	[67]
2		*C. bornmuelleri*	*α*-Glucosidase inhibition	Aerial parts	MeOH	10.17 ± 0.91 (mg ACAE/g extract)	[67]
3		*C. bornmuelleri*	*α*-Glucosidase inhibition	Aerial parts	Decoction	1.95 ± 0.07 (mg ACAE/g extract)	[67]
4		*C. bornmuelleri*	*α*-Glucosidase inhibition	Aerial parts	Infusion	2.36 ± 0.25 (mg ACAE/g extract)	[67]
5		*C. bornmuelleri*	*α*-Amylase inhibition	Aerial parts	Ethyl acetate	19.90 ± 0.89 (mg ACAE/g extract)	[67]
6		*C. bornmuelleri*	*α*-Amylase inhibition	Aerial parts	MeOH	16.73 ± 0.34 (mg ACAE/g extract)	[67]
7		*C. bornmuelleri*	*α*-Amylase inhibition	Aerial parts	Decoction	3.98 ± 0.22 (mg ACAE/g extract)	[67]
8		*C. bornmuelleri*	*α*-Amylase inhibition	Aerial parts	Infusion	3.54 ± 0.66 (mg ACAE/g extract)	[67]
9		*C. calcitrapa*	*α*-Glucosidase inhibition	Aerial parts	MeOH	4.38 ± 0.31 (mg/ml)	[68]
10		*C. centaurium*	*α*-Amylase inhibition	Roots	MeOH	32.51 ± 0.34%	[69]
11		*C. centaurium*	*α*-Amylase inhibition	Roots	Aqueous	—	[69]
12		*C. centaurium*	*α*-Amylase inhibition	Roots	Polyphenol	—	[69]
13		*C. centaurium*	*α*-Amylase inhibition	Roots	*n*-Hexane	158 (*μ*g/ml)	[69]
14		*C. depressa*	*α*-Glucosidase inhibition	Aerial parts	Ethyl acetate	46.11 ± 0.97%	[70]
15		*C. depressa*	*α*-Glucosidase inhibition	Aerial parts	Chloroform	53.45 ± 1.98%	[70]
16		*C. depressa*	*α*-Amylase inhibition	Aerial parts	Ethyl acetate	36.93 ± 0.97%	[70]
17		*C. depressa*	*α*-Amylase inhibition	Aerial parts	Chloroform	43.97 ± 0.92%	[70]
18		*C. drabifolia* subsp. *detonsa*	*α*-Glucosidase inhibition	Aerial parts	Ethyl acetate	43.10 ± 2.41%	[70]
19		*C. drabifolia* subsp. *detonsa*	*α*-Glucosidase inhibition	Aerial parts	Chloroform	36.03 ± 0.24%	[70]
20		*C. drabifolia* subsp. *detonsa*	*α*-Amylase inhibition	Aerial parts	Ethyl acetate	25.58 ± 0.38%	[70]
21		*C. drabifolia* subsp. *detonsa*	*α*-Amylase inhibition	Aerial parts	Chloroform	25.28 ± 0.38%	[70]
22		*C. fenzlii*	*α*-Glucosidase inhibition	Aerial parts	MeOH	0.331 (mmol ACAE/g dry weight)	[71]
23		*C. fenzlii*	*α*-Amylase inhibition	Aerial parts	MeOH	0.354 (mmol ACAE/g dry weight)	[71]
24		*C. hypoleuca*	*α*-Glucosidase inhibition	Flowers	EtOH	10.33 ± 0.04 (mmol ACAE/g extract)	[72]
25		*C. hypoleuca*	*α*-Glucosidase inhibition	Flowers	MeOH	12.77 ± 0.61 (mmol ACAE/g extract)	[72]
26		*C. hypoleuca*	*α*-Glucosidase inhibition	Flowers	Ethyl acetate	19.61 ± 0.05 (mmol ACAE/g extract)	[72]
27		*C. hypoleuca*	*α*-Glucosidase inhibition	Stems	EtOH	9.10 ± 0.06 (mmol ACAE/g extract)	[72]
28		*C. hypoleuca*	*α*-Glucosidase inhibition	Stems	MeOH	8.66 ± 0.08 (mmol ACAE/g extract)	[72]
29		*C. hypoleuca*	*α*-Glucosidase inhibition	Stems	Ethyl acetate	12.62 ± 0.21 (mmol ACAE/g extract)	[72]
30		*C. hypoleuca*	*α*-Amylase inhibition	Flowers	EtOH	82.65 ± 1.31 (mmol ACAE/g extract)	[72]
31		*C. hypoleuca*	*α*-Amylase inhibition	Flowers	MeOH	102.41 ± 1.18 (mmol ACAE/g extract)	[72]
32		*C. hypoleuca*	*α*-Amylase inhibition	Flowers	Ethyl acetate	106.72 ± 1.10 (mmol ACAE/g extract)	[72]
33		*C. hypoleuca*	*α*-Amylase inhibition	Stems	EtOH	63.64 ± 1.05 (mmol ACAE/g extract)	[72]
34		*C. hypoleuca*	*α*-Amylase inhibition	Stems	MeOH	66.66 ± 0.67 (mmol ACAE/g extract)	[72]
35		*C. hypoleuca*	*α*-Amylase inhibition	Stems	Ethyl acetate	72.41 ± 0.61 (mmol ACAE/g extract)	[72]
36		*C. karduchorum*	*α*-Glucosidase inhibition	Roots	Hydrophilic (80%EtOH, 19% H_2_O, and 1% of 0.1% trifluoroacetic acid, v/v/v)	5.35 ± 0.08 (mg/ml)	[73]
37		*C. karduchorum*	*α*-Glucosidase inhibition	Stems	Hydrophilic (80% ethanol, 19% H_2_O, and 1% of 0.1% trifluoroacetic acid, v/v/v)	1.42 ± 0.10 (mg/ml)	[73]
38		*C. karduchorum*	*α*-Glucosidase inhibition	Leaves	Hydrophilic (80% ethanol, 19% H_2_O, and 1% of 0.1% trifluoroacetic acid, v/v/v)	0.63 ± 0.00 (mg/ml)	[73]
39		*C. karduchorum*	*α*-Glucosidase inhibition	Flowers	Hydrophilic (80% ethanol, 19% H_2_O, and 1% of 0.1% trifluoroacetic acid, v/v/v)	1.51 ± 0.22 (mg/ml)	[73]
40		*C. karduchorum*	*α*-Amylase inhibition	Roots	Hydrophilic (80% ethanol, 19% H_2_O, and 1% of 0.1% trifluoroacetic acid, v/v/v)	Not active	[73]
41		*C. karduchorum*	*α*-Amylase inhibition	Stems	Hydrophilic (80% ethanol, 19% H_2_O, and 1% of 0.1% trifluoroacetic acid, v/v/v)	Not active	[73]
42		*C. karduchorum*	*α*-Amylase inhibition	Leaves	Hydrophilic (80% ethanol, 19% H_2_O, and 1% of 0.1% trifluoroacetic acid, v/v/v)	14.63 ± 0.67 (mg/ml)	[73]
43		*C. karduchorum*	*α*-Amylase inhibition	Flowers	Hydrophilic (80% ethanol, 19% H_2_O, and 1% of 0.1% trifluoroacetic acid, v/v/v)	Not active	[73]
44		*C. kotschyi* var. *persica*	*α*-Glucosidase inhibition	Aerial parts	Ethyl acetate	42.35 ± 2.22%	[70]
45		*C. kotschyi* var. *persica*	*α*-Glucosidase inhibition	Aerial parts	Chloroform	49.42 ± 0.92%	[70]
46		*C. kotschyi* var. p*ersica*	*α*-Amylase inhibition	Aerial parts	Ethyl acetate	36.16 ± 0.13%	[70]
47		*C. kotschyi* var. *persica*	*α*-Amylase inhibition	Aerial parts	Chloroform	42.72 ± 0.17%	[70]
48		*C. papposa*	*α*-Glucosidase inhibition	Aerial parts	Dichloromethane	227.6 ± 4.4 (*μ*g/ml)	[8]
49		*C. papposa*	*α*-Glucosidase inhibition	Aerial parts	Ethyl acetate	791.9 ± 1.8 (*μ*g/ml)	[8]
50		*C. papposa*	*α*-Glucosidase inhibition	Aerial parts	*n*-Butanol	Not active	[8]
51		*C. patula*	*α*-Glucosidase inhibition	Aerial parts	Ethyl acetate	54.88 ± 1.11%	[70]
52		*C. patula*	*α*-Glucosidase inhibition	Aerial parts	Chloroform	56.11 ± 0.24%	[70]
53		*C. patula*	*α*-Amylase inhibition	Aerial parts	Ethyl acetate	31.70 ± 0.04%	[70]
54		*C. patula*	*α*-Amylase inhibition	Aerial parts	Chloroform	33.30 ± 0.04%	[70]
55		*C. pulchella*	*α*-Glucosidase inhibition	Aerial parts	Ethyl acetate	35.59 ± 0.58%	[70]
56		*C. pulchella*	*α*-Glucosidase inhibition	Aerial parts	Chloroform	60.31 ± 2.13%	[70]
57		*C. pulchella*	*α*-Amylase inhibition	Aerial parts	Ethyl acetate	21.54 ± 0.04%	[70]
58		*C. pulchella*	*α*-Amylase inhibition	Aerial parts	Chloroform	59.54 ± 0.59%	[70]
59		*C. saligna*	*α*-Glucosidase inhibition	Leaves	Ethyl acetate	23.80 ± 0.06 (mmol ACAE/g extract)	[74]
60		*C. saligna*	*α*-Glucosidase inhibition	Leaves	MeOH	12.57 ± 1.97 (mmol ACAE/g extract)	[74]
61		*C. saligna*	*α*-Glucosidase inhibition	Leaves	Aqueous	3.32 ± 0.40 (mmol ACAE/g extract)	[74]
62		*C. saligna*	*α*-Amylase inhibition	Leaves	Ethyl acetate	0.80 ± 0.01 (mmol ACAE/g extract)	[74]
63		*C. saligna*	*α*-Amylase inhibition	Leaves	MeOH	0.59 ± 0.01 (mmol ACAE/g extract)	[74]
64		*C. saligna*	*α*-Amylase inhibition	Leaves	Aqueous	0.16 ± 0.01 (mmol ACAE/g extract)	[74]
65		*C. tchihacheffii*	*α*-Glucosidase inhibition	Aerial parts	Ethyl acetate	58.23 ± 0.53%	[70]
66		*C. tchihacheffii*	*α*-Glucosidase inhibition	Aerial parts	Chloroform	53.45 ± 1.40%	[70]
67		*C. tchihacheffii*	*α*-Amylase inhibition	Aerial parts	Ethyl acetate	29.89 ± 1.01%	[70]
68		*C. tchihacheffii*	*α*-Amylase inhibition	Aerial parts	Chloroform	40.26 ± 0.29%	[70]
69		*C. triumfettii*	*α*-Glucosidase inhibition	Aerial parts	Ethyl acetate	69.88 ± 1.16%	[70]
70		*C. triumfettii*	*α*-Glucosidase inhibition	Aerial parts	Chloroform	41.12 ± 0.77%	[70]
71		*C. triumfettii*	*α*-Amylase inhibition	Aerial parts	Ethyl acetate	42.84 ± 0.34%	[70]
72		*C. triumfettii*	*α*-Amylase inhibition	Aerial parts	Chloroform	22.40 ± 0.17%	[70]
73		*C. triumfettii*	*α*-Glucosidase inhibition	Stems	EtOH	3.74 ± 0.05 (mmol ACAE/g extract)	[14]
74		*C. triumfettii*	*α*-Glucosidase inhibition	Stems	MeOH	3.77 ± 0.05 (mmol ACAE/g extract)	[14]
75		*C. triumfettii*	*α*-Glucosidase inhibition	Stems	Ethyl acetate	4.13 ± 0.04 (mmol ACAE/g extract)	[14]
76		*C. triumfettii*	*α*-Glucosidase inhibition	Flowers	EtOH	2.27 ± 0.01 (mmol ACAE/g extract)	[14]
77		*C. triumfettii*	*α*-Glucosidase inhibition	Flowers	MeOH	2.09 ± 0.03 (mmol ACAE/g extract)	[14]
78		*C. triumfettii*	*α*-Glucosidase inhibition	Flowers	Ethyl acetate	1.42 ± 0.05 (mmol ACAE/g extract)	[14]
79		*C. triumfettii*	*α*-Amylase inhibition	Stems	EtOH	137.39 ± 0.76 (mmol ACAE/g extract)	[14]
80		*C. triumfettii*	*α*-Amylase inhibition	Stems	MeOH	127.57 ± 0.72 (mmol ACAE/g extract)	[14]
81		*C. triumfettii*	*α*-Amylase inhibition	Stems	Ethyl acetate	165.47 ± 0.72 (mmol ACAE/g extract)	[14]
82		*C. triumfettii*	*α*-Amylase inhibition	Flowers	EtOH	137.42 ± 0.75 (mmol ACAE/g extract)	[14]
83		*C. triumfettii*	*α*-Amylase inhibition	Flowers	MeOH	114.06 ± 0.50 (mmol ACAE/g extract)	[14]
84		*C. triumfettii*	*α*-Amylase inhibition	Flowers	Ethyl acetate	116.85 ± 0.85 (mmol ACAE/g extract)	[14]
85		*C. urvillei* subsp. *hayekiana*	*α*-Glucosidase inhibition	Aerial parts	Ethyl acetate	67.66 ± 0.05%	[70]
86		*C. urvillei* subsp. *hayekiana*	*α*-Glucosidase inhibition	Aerial parts	Chloroform	43.65 ± 0.39%	[70]
87		*C. urvillei* subsp. *hayekiana*	*α*-Amylase inhibition	Aerial parts	Ethyl acetate	43.20 ± 0.59%	[70]
88		*C. urvillei* subsp. *hayekiana*	*α*-Amylase inhibition	Aerial parts	Chloroform	17.53 ± 0.08%	[70]

89		*C. alexanderina*	Reduction of blood glucose level	Leaves	MeOH		[75]
90	*In vivo* studies	*C. aspera*	It exhibited an important hypoglycemic effect by oral route and chronic administration in diabetic rats; the extract obtained by exhaustion with hot water showed an acute hypoglycemic activity in normal animals	Flowers	Aqueous	—	[76]
91		*C. bruguierana*	The ethyl acetate extract resulted in the best reduction of blood glucose The aqueous extract resulted in the best reduction of PEPCK activity and increment in hepatic GP activity	Aerial fruiting parts	Aqueous, dichloromethane, ethyl acetate, and methanol	—	[77]
92		*C. corubionensis*	Consumption of aqueous extracts of leaves and flowers at the dose of 5 g/kg led to the reduction of blood glucose levels; aqueous extract of flowers (50 mg/ml) could increase insulin release from isolated islets of Langerhans	Leaves and flowers	Aqueous and EtOH	—	[78]
93		*C. horrida*	Reduction in blood glucose level in chronic and acute condition Using the extract significantly improved peripheral nerve function of diabetic mice *via* hot plate and tail flick tests	Herb and roots	MeOH	—	[79]

^a^IC_50_ values reported as mg/ml, *μ*g/ml, mmol ACAE/g extract, or inhibition percent (%). ^b^ACAE = acarbose equivalent.

**Table 2:** Chemical compounds isolated from *Centaurea* spp.

Entry	*Centaurea* spp.	Phytochemical constituents	References
1	*C. alexanderina*	Sesquiterpene lactones and flavonoids (kaempferol 3-*O*-rutinoside, rutin, apigenin 7-*O*-galacturonic acid methyl ester, apigenin 7-*O*-*β*-D-glucoside, astragalin, centaurein, vicenin, vitexin, isovitexin, kaempferol, apigenin, quercetin, jaceosidin, and nepetin)	[75, 104, 115, 116]

2	*C. aspera*	Sesquiterpene lactones (dehydromelitensin, melitensin, isomelitensin, eudesmanolides, and dihydrostenophyllolide) and flavonoids (6-methoxyluteolin (nepetin), 6-methoxyacacetin (pectolinarigenin), 6-methoxyapigenin (hispidulin), and 6-methoxychrisoeriol (jaceosidin)).	[52, 116–118]

3	*C. bornmuelleri*	Flavonoids (afzelin, astragalin, isorhamnetin, apigenin, quercetin, luteolin, and kaempferol), phenolic acids (caffeoylquinic acids and chlorogenic acid), sterol (stigmast-4-en-3gamma-ol), and lignans (arctiin, arctigenin, matairesinol, and matairesinoside)	[67, 92, 119]

4	*C. bruguierana*	Sesquiterpene lactones (cnicin and dehydromelitensin-8-acetate) and flavonoids (kaempferol, rutin, quercetin, cirsimaritin, cirsilinelol, and eupatilin)	[77, 104, 112, 113, 120–123]

5	C. calcitrapa	Sterols, sesquiterpene lactones and their closely related group of triterpenoids, lignans, flavonoids (apigenin, luteolin, scutellarein, chrysoeriol, nepetin, jaceosidin, eupatorin, kaempferol, kaempferide, jaceidin, and centaureidin), alkaloids (stizolphine and choline), and phenolic acids (derivatives of hydroxycinnamic acids: *p*-coumaric, ferulic, caffeic, and chlorogenic acid; derivatives of hydroxybenzoic acids: *p*-hydroxybenzoic, protocatechuic, gallic, and gentisic acid)	[124–132]

6	*C. centaurium*	Fatty acids (11, 14-eicosadienoic acid methyl ester, 9-octadecenoic acid methyl ester, and 9-octadecenoic acid) and terpenes (cypirene, *α*-zingiberene, *β*-farnesene, *β*-santalene, *β*-bisabolene, *β*-himachalene, and azulene)	[69]

7	*C. corubionensis*	Has not been fully characterized	

8	*C. depressa*	Phenolic compounds, condensed tannins, flavonoids (luteolin, kaempferol, scutellarein 7-*β*-D-glucuronoside, scutellarein 5-*β*-D-glucuronoside, quercetin, isoquercitrin, quereimeritrin, and apigenin), monoterpenoid (piperitone), sesquiterpenoid (elemol), and sesquiterpene lactones (solstitialin A and acetyl solstitialin)	[70, 90, 92, 133–137]

9	*C. drabifolia*	Flavonoids, sesquiterpene lactones (belonging to the guaiane class; centaurea lactone, cynaropicrin, aguerin B, 8*α*-isovaleryloxyzaluzanin C, 8*α*-acetoxyzaluzanin C, and 4*β*,15-dihydro-3-dehydrosolstitialin A), and phenolic compounds (protocatechuic acid, 5-caffeoylquinic acid, 5-feruloylquinic acid, orientin, vitexin, quercetin, quercetin-3-*O*-glucoside, patuletin-*O*-hexoside, luteolin, luteolin-7-*O*-rutinoside, luteolin-7-*O*-glucoside, isovitexin, apigenin, and hispidulin)	[138–142]

10	*C. fenzlii*	Flavonoids (cirsiliol, isorhamnetin, hispidulin, and cirsimaritin)	[95]

11	*C. horrida*	Flavonoids (horridin, apigenin, rutin, apigenin-3-Ο-glucuronide, kaempferol-3-*O*-glucuronide, apigenin-8-C-*α*-L-arabinoside, apigenin-6-C-*α*-L-arabinoside, apigenin-7-Ο-*β*-D-glucoside, apigenin6,8-di-C-*β*-D-glucoside, scutelarein-7-*O*-*β*-D-glucoside, kaempferol-3-*O*-*β*-D-glucoside, kaempferol-3-O-*α*-L- rhamnoside, vitexin, isovitexin, orientin, schaftoside, hispidulin, fisetin, quercetin, quercetin-3-*O*-*α*-L-rhamnoside, and quercetin-3-*O*-*β*-D-galactoside), lactones, phenolic acids, pentacyclic triterpenes, sterol glucoside, and Q acid derivatives	[104, 143–146]

12	*C. hypoleuca*	Sesquiterpene lactones (centaurepensin, acroptillin, cynaropicrin, janerin, linichlorin, and repin) and phenolic compound (catechin and chlorogenic acid)	[72, 126, 147–150]

13	*C. karduchorum*	Phenolic compounds (chlorogenic acid, apigenin, and luteolin glycosides)	[73, 150, 151]

14	*C. kotschyi*	Sesquiterpene lactones (germacrene D, *β*-caryophyllene, *β*-cedrene, *β*-bisabolene, and bicyclogermacrene), phenolic compounds, and flavonoid (patuletin-7-O-glucoside)	[70, 116, 152, 153]

15	*C. papposa*	Phenolic acids (quinic acid, malic acid, gallic acid, protocatechuic acid, chlorogenic acid, caffeic acid, ferulic acid, salicylic acid, vanillic acid, coumarin, syringic acid, apigenin, and apigetrin), flavonoids, and terpenes	[8, 154, 155]

16	*C. patula*	Phenolic compounds (protocatechuic acid, caffeic acid, 5-feruloylquinic acid, orientin, vitexin, patuletin-*O*-hexoside, luteolin-7-*O*-glucoside, isovitexin, quercetin, apigenin, hispidulin, and luteolin), sesquiterpenes (spathulenol), and diterpene alcohol (phytol)	[141, 156]

17	*C. pulchella*	Phenolics content, condensed tannins, and fatty acid (linoleic acid, *α*-linoleic acid, and palmitic acid)	[70]

18	*C. saligna*	Flavonoids (rutin, hesperidin, quercetin, luteolin, kaempferol, and apigenin) and phenolic compounds (rosmarinic acid and *p*-coumaric acid)	[74]

19	*C. tchihacheffii*	Phenolic compounds	[70]

20	*C. triumfettii*	Phenolic compounds (chlorogenic acid, ferulic acid, p-coumaric acid, and caffeic acid)	[14, 150]

21	*C. urvillei*	Flavonoids (naringenin-7-*O*-*β*-D glucuronopyranoside, 6-hydroxykaempferol-7-*O*-*β*-D glucuronopyranoside, hispidulin-7-*O*-*β*-D-glucuronopyranoside, apigenin-7-*O*-*β*-D-methylglucuronopyranoside, hispidulin-7-*O*-*β*-D-methylglucuronopyranoside, hispidulin-7-*O*-*β*-D-glucopyranoside, apigenin-7-*O*-*β*-D-glucopyranoside, kaempferol, apigenin, luteolin, eriodictyol-7-*O*-*β*-D-glucuronopyranoside, arbutin, salidroside, and 3,5-dihydroxyphenethyl alcohol-3-*O*-*β*-D-glucopyranoside)	[70, 92, 157]

## Data Availability

The data supporting this review are from the previously reported studies and data sets which have been cited. The data used to support the findings of this study are available from the corresponding author upon request.

## References

[1] Bell G. I. (1991). Molecular defects in diabetes mellitus. *Diabetes*.

[2] Wild S., Roglic G., Green A., Sicree R., King H. (2004). Global prevalence of diabetes: estimates for the year 2000 and projections for 2030. *Diabetes Care*.

[3] Bedekar A., Shah K., Koffas M. (2010). Natural products for type II diabetes treatment. *Advances in Applied Microbiology*.

[4] Cerf M. E. (2013). Beta cell dysfunction and insulin resistance. *Frontiers in Endocrinology*.

[5] Alam F., Shafique Z., Amjad S. T., Bin Asad M. H. H. (2019). Enzymes inhibitors from natural sources with antidiabetic activity: a review. *Phytotherapy Research*.

[6] Chehade J. M., Mooradian A. D. (2000). A rational approach to drug therapy of type 2 diabetes mellitus. *Drugs*.

[7] Kitada M., Zhang Z., Mima A., King G. L. (2010). Molecular mechanisms of diabetic vascular complications. *Journal of Diabetes Investigation*.

[8] Mawahib C., Nabila Z., Nabila S., Chawki B., Salah A. (2019). LC-MS analysis, antioxidant and alpha-glucosidase inhibitory activities of Centaurea papposa extracts. *Bangladesh Journal of Pharmacology*.

[9] Mukherjee P. K., Kumar V., Mal M., Houghton P. J. (2007). Acetylcholinesterase inhibitors from plants. *Phytomedicine*.

[10] Notkins A. L. (2002). Immunologic and genetic factors in type 1 diabetes. *Journal of Biological Chemistry*.

[11] Ahamad J., Naquvi K. J. (2011). Review on role of natural alpha-glucosidase inhibitors for management of diabetes mellitus. *International Journal of Biomedical Research*.

[12] Chiasson J.-L., Josse R. G., Gomis R., Hanefeld M., Karasik A., Laakso M. (2002). Acarbose for prevention of type 2 diabetes mellitus: the STOP-NIDDM randomised trial. *The Lancet*.

[13] Lasano N. F., Ramli N. S., Hamid A. H., Karim R., Pak Dek M. S., Shukri R. (2019). Effects of different extraction solvents on polyphenols and antioxidant capacity of peel, pulp and seed kernel of kuini (*Mangifera odorata*). *Oriental Pharmacy and Experimental Medicine*.

[14] Acet T. (2021). Determining the phenolic components by using HPLC and biological activity of *Centaurea triumfetti*. *Plant Biosystems—An International Journal Dealing with all Aspects of Plant Biology*.

[15] Tadera K., Minami Y., Takamatsu K., Matsuoka T. (2006). Inhibition of *α*-glucosidase and *α*-amylase by flavonoids. *Journal of Nutritional Science and Vitaminology*.

[16] Kumar R., Pate D. K., Prasad S. K., Sairam K., Hemalatha S. (2011). Antidiabetic activity of alcoholic leaves extract of *Alangium lamarckii* Thwaites on streptozotocin-nicotinamide induced type 2 diabetic rats. *Asian Pacific Journal of Tropical Medicine*.

[17] Bhat M., Kothiwale S. K, Tirmale A. R, Bhargava S. Y, Joshi B. N (2011). Antidiabetic properties of *Azardiracta indica* and *Bougainvillea spectabilis*: *in vivo* studies in murine diabetes model. *Evidence-based Complementary and Alternative Medicine*.

[18] Watcharachaisoponsiri T., Sornchan P., Charoenkiatkul S., Suttisansanee U. (2016). The *α*-glucosidase and *α*-amylase inhibitory activity from different chili pepper extracts. *International Food Research Journal*.

[19] Varghese G. K., Bose L. V., Habtemariam S. (2013). Antidiabetic components of *Cassia alata* leaves: identification through *α*-glucosidase inhibition studies. *Pharmaceutical Biology*.

[20] Jaiswal N., Bhatia V., Srivastava S. P., Srivastava A. K., Tamrakar A. K. (2012). Antidiabetic effect of *Eclipta alba* associated with the inhibition of alpha-glucosidase and aldose reductase. *Natural Product Research*.

[21] Suryanarayana P., Anil Kumar P., Saraswat M., Mark Petrash J., Bhanuprakash Reddy G. (2004). Inhibition of aldose reductase by tannoid principles of *Emblica officinalis*: implications for the prevention of sugar cataract. *Molecular Vision*.

[22] Kabir A. U., Samad M. B., Ahmed A. (2015). Aqueous fraction of *Beta vulgaris* ameliorates hyperglycemia in diabetic mice due to enhanced glucose stimulated insulin secretion, mediated by acetylcholine and GLP-1, and elevated glucose uptake via increased membrane bound GLUT4 transporters. *PLoS One*.

[23] Gulati V., Harding I. H., Palombo E. A. (2012). Enzyme inhibitory and antioxidant activities of traditional medicinal plants: potential application in the management of hyperglycemia. *BMC Complementary and Alternative Medicine*.

[24] Mopuri R., Islam M. S. (2016). Antidiabetic and anti-obesity activity of Ficus carica: *in vitro* experimental studies. *Diabetes & Metabolism*.

[25] Orhan N., Hoçbaç S., Orhan D. D., Asian M., Ergun F. (2014). Enzyme inhibitory and radical scavenging effects of some antidiabetic plants of Turkey. *Iranian Journal of Basic Medical Sciences*.

[26] Guo Z., Niu X., Xiao T., Lu J., Li W., Zhao Y. (2015). Chemical profile and inhibition of *α*-glycosidase and protein tyrosine phosphatase 1B (PTP1B) activities by flavonoids from licorice (*Glycyrrhiza uralensis* Fisch). *Journal of Functional Foods*.

[27] Chen G., Guo M. (2017). Rapid screening for *α*-glucosidase inhibitors from *Gymnema sylvestre* by affinity ultrafiltration-HPLC-MS. *Frontiers in Pharmacology*.

[28] Ha K.-S., Jo S.-H., Mannam V., Kwon Y.-I., Apostolidis E. (2016). Stimulation of phenolics, antioxidant and *α*-glucosidase inhibitory activities during barley (*Hordeum vulgare* L.) seed germination. *Plant Foods for Human Nutrition*.

[29] Lee S., Mediani A., Nur Ashikin A. H., Azliana A. B. S., Abas F. (2014). Antioxidant and *α*-glucosidase inhibitory activities of the leaf and stem of selected traditional medicinal plants. *International Food Research Journal*.

[30] Prashanth D., Amit A., Samiulla D. S., Asha M. K., Padmaja R. (2001). *α*-glucosidase inhibitory activity of *Mangifera indica* bark. *Fitoterapia*.

[31] Malapermal V., Botha I., Krishna S. B. N., Mbatha J. N. (2017). Enhancing antidiabetic and antimicrobial performance of *Ocimum basilicum*, and *Ocimum sanctum* (L.) using silver nanoparticles. *Saudi Journal of Biological Sciences*.

[32] Li Y., Wen S., Kota B. P. (2005). *Punica granatum* flower extract, a potent *α*-glucosidase inhibitor, improves postprandial hyperglycemia in Zucker diabetic fatty rats. *Journal of Ethnopharmacology*.

[33] Kato A., Higuchi Y., Goto H. (2006). Inhibitory effects of *Zingiber officinale* roscoe derived components on aldose reductase activity *in vitro* and *in vivo*. *Journal of Agricultural and Food Chemistry*.

[34] Aderogba M., Ndhlala A., Rengasamy K., Van Staden J. (2013). Antimicrobial and selected *in vitro* enzyme inhibitory effects of leaf extracts, flavonols and indole alkaloids isolated from *Croton menyharthii*. *Molecules*.

[35] Tiong S., Looi C., Hazni H. (2013). Antidiabetic and antioxidant properties of alkaloids from *Catharanthus roseus* (L.) G. Don. *Molecules*.

[36] Puneeth H. R., Sharada A. (2015). Antioxidant and hypoglycemic effects of curcumin pyrazole derivatives. *International Journal of Pharmacy and Pharmaceutical Sciences*.

[37] Nanumala S. K., Tulasi P., Sujitha E. (2015). In vitro anti‐diabetic activity of seed extracts of *Cassia auriculata* and *Cassia angustifolia*. *European Journal of Experimental Biology*.

[38] Shihabudeen H. M. S., Priscilla D. H., Thirumurugan K. (2011). Cinnamon extract inhibits *α*-glucosidase activity and dampens postprandial glucose excursion in diabetic rats. *Nutrition & Metabolism*.

[39] Meng Y., Su A., Yuan S. (2016). Evaluation of total flavonoids, myricetin, and quercetin from *Hovenia dulcis* Thunb. As inhibitors of *α*-amylase and *α*-glucosidase. *Plant Foods for Human Nutrition*.

[40] Ali H., Houghton P. J., Soumyanath A. (2006). *α*-amylase inhibitory activity of some Malaysian plants used to treat diabetes; with particular reference to *Phyllanthus amarus*. *Journal of Ethnopharmacology*.

[41] Yang Z., Wang Y., Wang Y., Zhang Y. (2012). Bioassay-guided screening and isolation of *α*-glucosidase and tyrosinase inhibitors from leaves of *Morus alba*. *Food Chemistry*.

[42] Chiang Y. C., Chen C. L., Jeng T. L., Sung J. M. (2014). In vitro inhibitory effects of cranberry bean (*Phaseolus vulgaris* L.) extracts on aldose reductase, *α*‐glucosidase and *α*‐amylase. *International Journal of Food Science & Technology*.

[43] de la Garza A. L., Etxeberria U., Lostao M. P. (2013). Helichrysum and grapefruit extracts inhibit carbohydrate digestion and absorption, improving postprandial glucose levels and hyperinsulinemia in rats. *Journal of Agricultural and Food Chemistry*.

[44] Çelik S., Rosselli S., Maggio A. M. (2006). Guaianolides and lignans from the aerial parts of *Centaurea ptosimopappa*. *Biochemical Systematics and Ecology*.

[45] Shoeb M., MacManus S. M., Jaspars M. (2006). Montamine, a unique dimeric indole alkaloid, from the seeds of *Centaurea montana* (Asteraceae), and its *in vitro* cytotoxic activity against the CaCo2 colon cancer cells. *Tetrahedron*.

[46] Flamini G., Pardini M., Morelli I. (2002). Flavonoid glycosides from *Centaurea pseudoscabiosa* subsp. *pseudoscabiosa* from Turkey. *Phytochemistry*.

[47] Honda G., Yeşilada E., Tabata M. (1996). Traditional medicine in Turkey VI. Folk medicine in West Anatolia: Afyon, Kütahya, Denizli, Mugla, Aydin provinces. *Journal of Ethnopharmacology*.

[48] Mozaffarian V. A. (2013). *Identification of Medicinal and Aromatic Plants of Iran*.

[49] Ansari A. A., Gill S. S., Zahid A., Naeem M. (2016). *Plant Biodiversity: Monitoring, Assessment and Conservation*.

[50] Kaij-a-Kamb M., Amoros M., Girre L. (1992). The chemistry and biological activity the the genus Centaurea. *Pharmaceutica Acta Helvetiae*.

[51] Yesilada E., Gürbüz I, Bedir E, Tatli I, Khan I. A (2004). Isolation of anti-ulcerogenic sesquiterpene lactones from *Centaurea solstitialis* L. ssp. solstitialis through bioassay-guided fractionation procedures in rats. *Journal of Ethnopharmacology*.

[52] Marco J. A., Sanz-Cervera J. F., Yuste A., Sancenón F., Carda M. (2005). Sesquiterpenes from *Centaurea aspera*. *Phytochemistry*.

[53] Robles M., Wang N., Kim R., Choi B. H. (1997). Cytotoxic effects of repin, a principal sesquiterpene lactone of Russian knapweed. *Journal of Neuroscience Research*.

[54] Flamini G., Pardini M., Morelli I. (2001). A flavonoid sulphate and other compounds from the roots of *Centaurea bracteata*. *Phytochemistry*.

[55] Shoeb M., Celik S., Jaspars M. (2005). Isolation, structure elucidation and bioactivity of schischkiniin, a unique indole alkaloid from the seeds of *Centaurea schischkinii*. *Tetrahedron*.

[56] Amigo J.-M., Debaerdemaeker T., Seoane E., Tortajada A., Picher M.-T. (1984). Structure and stereochemistry of stenophyllolide, a germacrolide from *Centaurea aspera* var. stenophylla. *Phytochemistry*.

[57] Öksüz S., Ayyildiz H. (1986). Sesquiterpene lactones from *Centaurea coronopifolia*. *Phytochemistry*.

[58] Ayad R., Akkal S. (2019). Phytochemistry and biological activities of algerian *Centaurea* and related genera. *Studies in Natural Products Chemistry*.

[59] Hussain Z., Waheed A., Qureshi R. A. (2004). The effect of medicinal plants of Islamabad and Murree region of Pakistan on insulin secretion from INS-1 cells. *Phytotherapy Research*.

[60] Özuslu E. (2005). Sof Dağı (Gaziantep) yöresindeki bazı bitkilerin etnobotanik özellikleri ve mahalli adları. *Kırsal Çevre Yıllığı*.

[61] Kıran Ö. (2006). Kozan yöresi florasindaki tibbi bitkiler ve bunlarin halk tibbinda kullanılışı.

[62] Gençay A. (2007). Cizre (Şırnak)’nin etnobotanik özellikleri.

[63] Durmuskahya C., Ozturk M. (2013). Ethnobotanical survey of medicinal plants used for the treatment of diabetes in Manisa, Turkey. *Sains Malaysiana*.

[64] Çakılcıoğlu U., Türkoğlu İ., Kürşat M. (2007). Harput (Elazığ) ve çevresinin etnobotanik özellikleri. *Doğu Anadolu Bölgesi Araştırmaları*.

[65] Şenguuml M. (2010). An ethnobotanical survey of medicinal plants of Yazıkonak and Yurtbaşı districts of Elazığ province, Turkey. *Journal of Medicinal Plants Research*.

[66] Ozturk M., Altay V., Latiff A. (2018). A comparative analysis of the medicinal plants used for diabetes mellitus in the traditional medicine in Turkey, Pakistan, and Malaysia. *Plant and Human Health*.

[67] Zengin G., Llorent-Martínez E. J., Sinan K. I., Yıldıztugay E., Picot-Allain C., Mahomoodally M. F. (2019). Chemical profiling of *Centaurea bornmuelleri* Hausskn. aerial parts by HPLC-MS/MS and their pharmaceutical effects: from nature to novel perspectives. *Journal of Pharmaceutical and Biomedical Analysis*.

[68] Kaskoos R. A. (2013). In-vitro *α*-glucosidase inhibition and antioxidant activity of methanolic extract of *Centaurea calcitrapa* from Iraq. *American Journal of Essential Oils and Natural Products*.

[69] Conforti F., Menichini F., Loizzo M. R. (2008). Antioxidant, *α*-amylase inhibitory and brine-shrimp toxicity studies on *Centaurea centaurium* L. methanolic root extract. *Natural Product Research*.

[70] Zengin G., Locatelli M., Carradori S., Mocan A. M., Aktumsek A. (2016). Total phenolics, flavonoids, condensed tannins content of eight centaurea species and their broad inhibitory activities against cholinesterase, tyrosinase, *α*-amylase and *α*-glucosidase. *Notulae Botanicae Horti Agrobotanici Cluj-Napoca*.

[71] Yirtici Ü. (2019). *Centaurea fenzlii* reichardt özütünün antioksidan özellikleri ve enzim inhibisyon etkisinin belirlenmesi. *Bitlis Eren Üniversitesi Fen Bilimleri Dergisi.*.

[72] Özcan K., Acet T., Çorbacı C. (2019). Centaurea hypoleuca DC: phenolic content, antimicrobial, antioxidant and enzyme inhibitory activities. *South African Journal of Botany*.

[73] Dalar A., Uzun Y., Mukemre M., Turker M., Konczak I. (2015). *Centaurea karduchorum* Boiss. from Eastern Anatolia: phenolic composition, antioxidant and enzyme inhibitory activities. *Journal of Herbal Medicine*.

[74] Zengin G., Bulut G., Mollica A., Nancy Picot-Allain C. M., Mahomoodally M. F. (2018). In vitro and in silico evaluation of *Centaurea saligna* (K.Koch) Wagenitz-an endemic folk medicinal plant. *Computational Biology and Chemistry*.

[75] Kubacey T. M., Haggag E. G., El-Toumy S. A., Ahmed A. A., El-Ashmawy I. M., Youns M. M. (2012). Biological activity and flavonoids from *Centaurea alexanderina* leaf extract. *Journal of Pharmacy Research*.

[76] Masso J., Adzet T. (1976). Hypoglycaemic activity of *Centaurea aspera* L (author’s transl). *Revista espanola de Fisiologia*.

[77] Khanavi M., Taheri M., Rajabi A. (2012). Stimulation of hepatic glycogenolysis and inhibition of gluconeogenesis are the mechanisms of antidiabetic effect of *Centaurea bruguierana* ssp. belangerana. *Asian Journal of Animal and Veterinary Advances*.

[78] Chuclá M., Lamela M., Gato A., Cadavid I. (1988). Centaurea corcubionensis: a study of its hypoglycemic activity in rats. *Planta Medica*.

[79] Raafat K., Boukhary R., Aboul-Ela M., El-Lakany A. (2013). Endogenous lebanese plants treating diabetes and related complications. *Natural Products Chemistry and Research*.

[80] Sarker S. D., Kumarasamy Y., Shoeb M. (2005). Antibacterial and antioxidant activities of three Turkish species of the genus Centaurea. *Oriental Pharmacy and Experimental Medicine*.

[81] Sarker S. D., Shoeb M., Celik S. (2007). Extracts of *Centaurea bornmuelleri* and Centaurea huber-morathii inhibit the growth of colon cancer cells *in vitro*. *Oriental Pharmacy and Experimental Medicine*.

[82] Hänsel R., Keller K., Rimpler H., Schneider G. (1994). *Hagers Handbuch der Pharmazeutischen Praxis: Drogen PZ*.

[83] List P. H., Hörhammer L. (1969). *Hagers Handbuch der Pharmazeutischen Praxis: Für Apotheker, Arzneimittelhersteller, Ärzte und Medizinalbeamte: Wirkstoffgruppen II Chemikalien und Drogen (A-AL)*.

[84] Csupor D., Blazsó G., Balogh Á., Hohmann J. (2010). The traditional Hungarian medicinal plant *Centaurea sadleriana* Janka accelerates wound healing in rats. *Journal of Ethnopharmacology*.

[85] Toribio M. S., Oriani S. D., Skliar M. I. (2004). Actividad antimicrobiana de *Centaurea solstitialis* y *Centaurea calcitrapa*. *Ars Pharmaceutica*.

[86] Soumia K., Tahar D., Lamari L. (2014). Antioxidant and antimicrobial activities of selected medicinal plants from Algeria. *Journal of Coastal Life Medicine*.

[87] Moghannem S. A., Sherbiny G. M. E., Sharaf M. H. (2016). Antibacterial activity of medicinal plant (*Centauraea calcitrapa*) against multi-drug resistant bacteria (MDRB). *The Asia Journal of Applied Microbiology*.

[88] Trabsa H., Baghiani A., Boussoualim N., Krache I., Arrar L. (2020). The *in vivo* and *in vitro* antioxidant and anti-hemolytic effect of Algerian *Centaurea calcitrapa* L. extracts. *Journal of Drug Delivery and Therapeutics*.

[89] Zengin G., Aktumsek A., Guler G. O., Cakmak Y. S., Yildiztugay E. (2011). Antioxidant properties of methanolic extract and fatty acid composition of *Centaurea urvillei* DC. subsp. hayekiana Wagenitz. *Records of Natural Products*.

[90] Akkol E. K., Arif R., Ergun F., Yesilada E. (2009). Sesquiterpene lactones with antinociceptive and antipyretic activity from two Centaurea species. *Journal of Ethnopharmacology*.

[91] Tekeli Y., Zengin G., Aktumsek A., Sezgin M., Torlak E. (2011). Antibacterial activities of extracts from twelve Centaurea species from Turkey. *Archives of Biological Sciences*.

[92] Khammar A., Djeddi S. (2012). Pharmacological and biological properties of some Centaurea species. *European Journal of Scientific Research*.

[93] Ernst E. (2005). The efficacy of herbal medicine—an overview. *Fundamental and Clinical Pharmacology*.

[94] Yirtici U., Yilmaz F., Serim G. (2012). The cytotoxic effect of endemic *Centaurea fenzlii* Reichardt on colon cancer cell lines. *Planta Medica*.

[95] Yirtici Ü., Göger F., Sarimahmut M., Ergene A. (2017). Cytotoxic and apoptotic effects of endemic *Centaurea fenzlii* Reichardt on the MCF-7 breast cancer cell line. *Turkish Journal of Biology*.

[96] Dalar A., Konczak I. (2012). Botanicals from Eastern Anatolia Region of Turkey: antioxidant capacity and phenolic constituents of endemic herbal medicines. *Journal of Herbal Medicine*.

[97] Adisakwattana S., Lerdsuwankij O., Poputtachai U., Minipun A., Suparpprom C. (2011). Inhibitory activity of cinnamon bark species and their combination effect with acarbose against intestinal *α*-glucosidase and pancreatic *α*-amylase. *Plant Foods for Human Nutrition*.

[98] Altundag E., Ozturk M. (2011). Ethnomedicinal studies on the plant resources of east Anatolia, Turkey. *Procedia—Social and Behavioral Sciences*.

[99] Keser S., Keser F., İsmail T. (2020). In vitro biological evaluation and phytochemical contents of three Centaurea L. species growing from Eastern Anatolia in Turkey. *Kahramanmaraş Sütçü İmam Üniversitesi Tarım Ve Doğa Dergisi*.

[100] Sönmez P. E. S., Çakilcioğlu U. (2020). Screening of antimicrobial effect against microorganisms threatening to human health of the endemic plant; *Centaurea saligna* (C. Koch) Wagenitz from Turkey. *Türk Doğa Ve Fen Dergisi*.

[101] Ozüdoğru B., Akaydın G, Erik S, Yesilada E (2011). Inferences from an ethnobotanical field expedition in the selected locations of Sivas and Yozgat provinces (Turkey). *Journal of Ethnopharmacology*.

[102] Turker H., Yıldırım A. B. (2015). Screening for antibacterial activity of some Turkish plants against fish pathogens: a possible alternative in the treatment of bacterial infections. *Biotechnology & Biotechnological Equipment*.

[103] El Sohafy S. M., Alqasoumi S. I., Metwally A. M. (2013). Evaluation of the hepatoprotective activity of some plants belonging to the tribe Cynareae growing in Egypt. *Journal of Medicinal Plants Research*.

[104] Abu-Odeh A. M., Talib W. H. (2021). Middle East medicinal plants in the treatment of diabetes: a review. *Molecules*.

[105] Said G. (2007). Diabetic neuropathy-a review. *Nature Clinical Practice Neurology*.

[106] Tesfaye S., Boulton A. J. M., Dyck P. J. (2010). Diabetic neuropathies: update on definitions, diagnostic criteria, estimation of severity, and treatments. *Diabetes Care*.

[107] Chiocchio I., Mandrone M., Sanna C., Maxia A., Tacchini M., Poli F. (2018). Screening of a hundred plant extracts as tyrosinase and elastase inhibitors, two enzymatic targets of cosmetic interest. *Industrial Crops and Products*.

[108] Naveen Y. P., Urooj A., Byrappa K. (2021). A review on medicinal plants evaluated for anti-diabetic potential in clinical trials: present status and future perspective. *Journal of Herbal Medicine*.

[109] Rahimi-Madiseh M., Naimi A., Nasri H., Rafieian-Kopaei M. (2016). Biochemical and histopathological changes in kidney of diabetic rats treated with hydroalcoholic extract of *Centaurea cyanus*. *Journal of Mazandaran University of Medical Sciences*.

[110] Shoeb M., MacManus S. M., Jaspars M. (2007). Bioactivity of two Turkish endemic Centaurea species, and their major constituents. *Revista Brasileira de Farmacognosia*.

[111] Janackovic P., Tesevic V., Marin P. D. (2008). Brine shrimp lethality bioassay of selected Centaurea L. species (Asteraceae). *Archives of Biological Sciences*.

[112] Ostad S. N., Rajabi A., Khademi R. (2016). Cytotoxic potential of *Centaurea bruguierana* ssp. belangerana: the MTT assay. *Acta Medica Iranica*.

[113] Nasr F. A., Shahat A. A., Alqahtani A. S. (2020). Centaurea bruguierana inhibits cell proliferation, causes cell cycle arrest, and induces apoptosis in human MCF-7 breast carcinoma cells. *Molecular Biology Reports*.

[114] Erol-Dayi Ö., Pekmez M., Bona M., Aras-Perk A., Arda N. (2011). Total phenolic contents, antioxidant activities cytotoxicity of three Centaurea species: *C. calcitrapa* subsp. *calcitrapa*, *C. ptosimopappa C. spicata*. *Free Radicals and Antioxidants*.

[115] Mosharrafa S., Mansour R. M. A., Abou-Zaid M., Saleh N. A. M. (1994). Some biologically active flavonoids from Egyptian members of the Compositae. *Bulletin of the Chemical Society of Ethiopia*.

[116] Louaar S., Achouri A, Lefahal M (2011). Flavonoids from Algerian endemic Centaurea microcarpa and their chemotaxonomical significance. *Natural Product Communications*.

[117] Cardona M. L., Fernández I., Pedro J. R., Pérez B. (1991). Sesquiterpene lactones and flavonoids from *Centaurea aspera*. *Phytochemistry*.

[118] Ferreres F., Tomas F., Guirado A., Tomas F. A. (1980). Agliconas de flavonoides en la *Centaurea aspera* (compositae). *Afinidad*.

[119] Shoeb A. (2007). Lignans and flavonoids from the seeds of *Centaurea bornmuelleri* Hausskn. Ex. Bornm and *Centaurea huber-morathii* Wagenitz. *Polish Journal of Chemistry*.

[120] Harraz F., Kassem F., El-Shaer N. (1994). Sesquiterpene lactones and flavonoids from *Centaurea bruguierana*. *Alexandria Journal of Pharmaceutical Sciences*.

[121] Mirzahosseini G., Manayi A., Khanavi M. (2019). Bio-guided isolation of *Centaurea bruguierana* subsp. belangerana cytotoxic components. *Natural Product Research*.

[122] Rustaiyan A., Niknejad A., Aynehchi Y. (1982). Chemical constituents of *Centaurea brugueriana*. *Planta Medica*.

[123] Lee K.-H. (1999). Novel antitumor agents from higher plants. *Medicinal Research Reviews*.

[124] Karawya M. (1975). Phytochemical study of *Centaurea calcitrapa* L. growing in Egypt. *Egyptian Journal of Pharmaceutical Sciences*.

[125] Dawidar A. (1989). Chemical constituents of two Centaurea species. *Pharmazie*.

[126] Easa A., Rizk A. (1992). Constituents of Centaurea species. *Qatar University Science Journal*.

[127] Marco J. A., Sanz J. F., Sancenon F., Susanna A., Rustaiyan A., Saberi M. (1992). Sesquiterpene lactones and lignans from Centaurea species. *Phytochemistry*.

[128] Formisano C., Rigano D., Senatore F. (2012). Flavonoids in subtribe Centaureinae (Cass.) Dumort.(Tribe Cardueae, Asteraceae): distribution and 13C-NMR spectral data. *Chemistry & Biodiversity*.

[129] Jafri H., Khan M. S. A., Ahmad I. (2019). In vitro efficacy of eugenol in inhibiting single and mixed-biofilms of drug-resistant strains of *Candida albicans* and *Streptococcus mutans*. *Phytomedicine*.

[130] Kitouni R., Benayache F., Benayache S. (2015). Flavonoids of the exudate of *Centaurea calcitrapa*. *Chemistry of Natural Compounds*.

[131] Ahmed Z., Hammouda F., Rizk A., Ismail S. (1970). Phytochemical studies of certain Centaurea species. *Planta Medica*.

[132] Dimkić I., Petrović M, Gavrilović M (2020). New perspectives of purple starthistle (*Centaurea calcitrapa*) leaf extracts: phytochemical analysis, cytotoxicity and antimicrobial activity. *AMB Express*.

[133] Bandyukova V. A., Khalmatov K. K., Alimov K. I. (1969). Flavonoids of *Centaurea depressa*. *Chemistry of Natural Compounds*.

[134] Hosseinimehr S. J., Pourmorad F, Shahabimajd N, Shahrbandy K, Hosseinzadeh R (2007). In vitro antioxidant activity of polygonium hyrcanicum, *Centaurea depressa*, *Sambucus ebulus*, *Mentha spicata* and *Phytolacca americana*. *Pakistan Journal of Biological Sciences: PJBS*.

[135] Esmaeili A., Panahi Z. A., Ebrahimzadeh M. A. (2014). Investigation of phytochemistry of gene of Centaurea grown in Iran. *Journal of Essential Oil Bearing Plants*.

[136] Karamenderes C., Konyalioglu S., Khan S., Khan I. A. (2007). Total phenolic contents, free radical scavenging activities and inhibitory effects on the activation of NF‐kappa B of eight Centaurea L. species. *Phytotherapy Research*.

[137] Boğa M. (2016). Phytochemical profile and some biological activities of three Centaurea species from Turkey. *Tropical Journal of Pharmaceutical Research*.

[138] Formisano C., Sirignano C., Rigano D. (2017). Antiproliferative activity against leukemia cells of sesquiterpene lactones from the Turkish endemic plant *Centaurea drabifolia* subsp. Detonsa. *Fitoterapia*.

[139] Kasapoğlu K. N., Altin G., Ahmad Farooqi A. (2020). Anti-proliferative, genotoxic and cytotoxic effects of phytochemicals isolated from Anatolian medicinal plants. *Cellular and Molecular Biology*.

[140] De Cicco P. (2020). Inhibitory effects of cynaropicrin on human melanoma progression by targeting MAPK, NF‐*κ*B, and Nrf‐2 signaling pathways *in vitro*. *Phytotherapy Research*.

[141] Zengin G., Zheleva-Dimitrova D., Gevrenova R., Aktumsek A., Sinan K. I., Mahomoodally M. F. (2019). A comparative assessment of the LC-MS profiles and cluster analysis of four Centaurea species from Turkey. *Biocatalysis and Agricultural Biotechnology*.

[142] Zengin G., Zheleva-Dimitrova D., Gevrenova R. (2018). Identification of phenolic components via LC-MS analysis and biological activities of two Centaurea species: *C. drabifolia* subsp. Drabifolia and *C. lycopifolia*. *Journal of Pharmaceutical and Biomedical Analysis*.

[143] Flamini G., Bulleri C., Morelli I. (2002). Secondary constituents from *Centaurea horrida* and their evolutionary meaning. *Biochemical Systematics and Ecology*.

[144] Flamini G., Bulleri C., Morelli I., Manunta A. (2000). A new flavonoid glycoside from *Centaurea horrida*. *Journal of Natural Products*.

[145] Boukhary R., Aboul-Ela M., Al-Hanbali O., El-Lakany A. (2017). Phenolic compounds from *Centaurea horrida* L. growing in Lebanon. *IJPPR*.

[146] Grafakou M.-E., Djeddi S., Tarek H., Skaltsa H. (2018). Secondary metabolites from the aerial parts of *Centaurea papposa* (Coss.) Greuter. *Biochemical Systematics and Ecology*.

[147] Mandel S., Youdim M. B. H. (2004). Catechin polyphenols: neurodegeneration and neuroprotection in neurodegenerative diseases. *Free Radical Biology and Medicine*.

[148] Zhang L., Chang C., Liu Y., Chen Z. m. (2011). Effect of chlorogenic acid on disordered glucose and lipid metabolism in db/db mice and its mechanism. *Acta Academiae Medicinae Sinicae*.

[149] Fu X., Lyu X., Liu H. (2019). Chlorogenic acid inhibits BAFF expression in collagen-induced arthritis and human synoviocyte MH7A cells by modulating the activation of the NF-*κ*B signaling pathway. *Journal of Immunology Research*.

[150] Meng S., Cao J., Feng Q., Peng J., Hu Y. (2013). Roles of chlorogenic acid on regulating glucose and lipids metabolism: a review. *Evidence-Based Complementary and Alternative Medicine*.

[151] Chen C.-Y., Peng W. H., Tsai K. D, Hsu S. L. (2007). Luteolin suppresses inflammation-associated gene expression by blocking NF-*κ*B and AP-1 activation pathway in mouse alveolar macrophages. *Life Sciences*.

[152] Oksuz S., Putun E. (1987). Flavonoids of centaurea kotschyi var kotschyi. *DOGA Turk Kimya Derg*.

[153] Ertugrul K., Dura H., Tugay O., Flamini G., Cioni P. L., Morelli I. (2003). Essential oils from flowers of *Centaurea kotschyi* var. kotschyi and *C. kotschyi* var. decumbens from Turkey. *Flavour and Fragrance Journal*.

[154] Ouattara L. H., Kabran G. R., Brice Kadja A., Bosson Tano M., Mamyrbekova-Békro J. A., Békro Y. (2016). Phytochemical survey and antioxidant activity of plant extracts from Côte D’ivoire used in traditional treatment of hemorrhoids. *International Journal of Innovation and Applied Studies*.

[155] Souilah N. N. (2021). Biochemical properties and *in vitro* activities of extracts from two Asteraceae endemic species wild (Algeria). *RHAZES: Green and Applied Chemistry*.

[156] Zengin G., Aktumsek A., Boga M., Ceylan R., Uysal S. (2016). Essential oil composition of an uninvestigated Centaurea species from Turkey: *Centaurea patula* DC. *Journal of Essential Oil Bearing Plants*.

[157] Gülcemal D., Alankuş-Çalışkan Ö., Karaalp C., Örs A. U., Ballar P., Bedir E. (2010). Phenolic glycosides with antiproteasomal activity from *Centaurea urvillei* DC. subsp. *urvillei*. *Carbohydrate Research*.

[158] Chen L., Lu X., El-Seedi H., Teng H. (2019). Recent advances in the development of sesquiterpenoids in the treatment of type 2 diabetes. *Trends in Food Science & Technology*.

[159] Gutiérrez R. M. P., Ramirez A. M. (2016). Hypoglycemic effects of sesquiterpene lactones from *Byrsonima crassifolia*. *Food Science and Biotechnology*.

[160] Luyen N. T., Tram L. H., Hanh T. T. H. (2013). Inhibitors of *α*-glucosidase, *α*-amylase and lipase from *Chrysanthemum morifolium*. *Phytochemistry Letters*.

[161] Chen X.-W., Liu W.-T., Wang Y.-X. (2016). Cyclopropanyldehydrocostunolide LJ attenuates high glucose-induced podocyte injury by suppressing RANKL/RANK-mediated NF-*κ*B and MAPK signaling pathways. *Journal of Diabetes and Its Complications*.

[162] Jia Q.-Q., Wang J.-C., Long J. (2013). Sesquiterpene lactones and their derivatives inhibit high glucose-induced NF-*κ*B activation and MCP-1 and TGF-*β*1 expression in rat mesangial cells. *Molecules*.

[163] Basha R. H., Sankaranarayanan C. (2016). *β*-Caryophyllene, a natural sesquiterpene lactone attenuates hyperglycemia mediated oxidative and inflammatory stress in experimental diabetic rats. *Chemico-Biological Interactions*.

[164] Basha R. H., Sankaranarayanan C. (2015). Protective role of *β*-caryophyllene, a sesquiterpene lactone on plasma and tissue glycoprotein components in streptozotocin-induced hyperglycemic rats. *Journal of Acute Medicine*.

[165] Aguilar-Ávila D. S., Flores-Soto M. E., Tapia-Vázquez C., Pastor-Zarandona O. A., López-Roa R. I., Viveros-Paredes J. M. (2019). *β*-Caryophyllene, a natural sesquiterpene, attenuates neuropathic pain and depressive-like behavior in experimental diabetic mice. *Journal of Medicinal Food*.

[166] Yang J., Wang X., Zhang C. (2021). Comparative study of inhibition mechanisms of structurally different flavonoid compounds on *α*-glucosidase and synergistic effect with acarbose. *Food Chemistry*.

[167] Zeng L., Zhang G., Lin S., Gong D. (2016). Inhibitory mechanism of apigenin on *α*-glucosidase and synergy analysis of flavonoids. *Journal of Agricultural and Food Chemistry*.

[168] Esmaeili M. A., Sadeghi H. (2009). Pancreatic Β-cell protective effect of rutin and apigenin isolated from *Teucrium polium*. *Pharmacology Online*.

[169] Jung U., Cho Y.-Y., Choi M.-S. (2016). Apigenin ameliorates dyslipidemia, hepatic steatosis and insulin resistance by modulating metabolic and transcriptional profiles in the liver of high-fat diet-induced obese mice. *Nutrients*.

[170] Barky A., Ezz A., Mohammed T. (2020). The potential role of apigenin in diabetes mellitus. *International Journal of Clinical Case Reports and Reviews*.

[171] Abu Bakar A. R., Manaharan T., Merican A. F., Mohamad S. B. (2018). Experimental and computational approaches to reveal the potential of *Ficus deltoidea* leaves extract as *α*-amylase inhibitor. *Natural Product Research*.

[172] Ni M., Hu X., Gong D., Zhang G. (2020). Inhibitory mechanism of vitexin on *α*-glucosidase and its synergy with acarbose. *Food Hydrocolloids*.

[173] Ganesan K., Ramkumar K. M., Xu B. (2020). Vitexin restores pancreatic *β*-cell function and insulin signaling through Nrf2 and NF-*κ*B signaling pathways. *European Journal of Pharmacology*.

[174] Wang Y., Alkhalidy H., Luo J., Liu D. (2019). Antidiabetic effects of hispidulin in streptozotocin‐induced insulin deficient mice. *The FASEB Journal*.

[175] Peng X., Zhang G., Liao Y., Gong D. (2016). Inhibitory kinetics and mechanism of kaempferol on *α*-glucosidase. *Food Chemistry*.

[176] Yin P., Yang L., Xue Q. (2018). Identification and inhibitory activities of ellagic acid- and kaempferol-derivatives from Mongolian oak cups against *α*-glucosidase, *α*-amylase and protein glycation linked to type II diabetes and its complications and their influence on HepG2 cells’ viability. *Arabian Journal of Chemistry*.

[177] Alkhalidy H., Moore W., Wang A. (2018). Kaempferol ameliorates hyperglycemia through suppressing hepatic gluconeogenesis and enhancing hepatic insulin sensitivity in diet-induced obese mice. *The Journal of Nutritional Biochemistry*.

[178] Sharma D., Kumar Tekade R., Kalia K. (2020). Kaempferol in ameliorating diabetes-induced fibrosis and renal damage: an *in vitro* and *in vivo* study in diabetic nephropathy mice model. *Phytomedicine*.

[179] Habtemariam S. (2011). *α*-glucosidase inhibitory activity of kaempferol-3-O-rutinoside. *Natural Product Communications*.

[180] Rey D., Miranda Sulis P., Alves Fernandes T. (2019). Astragalin augments basal calcium influx and insulin secretion in rat pancreatic islets. *Cell Calcium*.

[181] Ghorbani A. (2017). Mechanisms of antidiabetic effects of flavonoid rutin. *Biomedicine & Pharmacotherapy*.

[182] Kim J.-S., Kwon C.-S., Son K. H. (2000). Inhibition of alpha-glucosidase and amylase by luteolin, a flavonoid. *Bioscience, Biotechnology, and Biochemistry*.

[183] Ouyang Z., Li W., Meng Q. (2017). A natural compound jaceosidin ameliorates endoplasmic reticulum stress and insulin resistance via upregulation of SERCA2b. *Biomedicine & Pharmacotherapy*.

[184] Park E., Kwon B.-M., Jung I.-K., Kim J.-H. (2014). Hypoglycemic and antioxidant effects of jaceosidin in streptozotocin-induced diabetic mice. *Journal of Nutrition and Health*.

[185] Pu P. (2016). Protection mechanisms of hesperidin on mouse with insulin resistance. *China Journal of Chinese Materia Medica*.

[186] Hanchang W., Khamchan A., Wongmanee N., Seedadee C. (2019). Hesperidin ameliorates pancreatic *β*-cell dysfunction and apoptosis in streptozotocin-induced diabetic rat model. *Life Sciences*.

[187] Kim J.-H., Kang M.-J., Choi H.-N., Jeong S.-M., Lee Y.-M., Kim J.-I. (2011). Quercetin attenuates fasting and postprandial hyperglycemia in animal models of diabetes mellitus. *Nutrition Research and Practice*.

[188] Arias N., Macarulla M. T., Aguirre L., Martínez-Castaño M. G., Portillo M. P. (2014). Quercetin can reduce insulin resistance without decreasing adipose tissue and skeletal muscle fat accumulation. *Genes & Nutrition*.

[189] Ishikawa A., Yamashita H., Hiemori M. (2007). Characterization of inhibitors of postprandial hyperglycemia from the leaves of *Nerium indicum*. *Journal of Nutritional Science and Vitaminology*.

[190] Jo S., Ka E., Lee H. (2009). Comparison of antioxidant potential and rat intestinal *α*-glucosidases inhibitory activities of quercetin, rutin, and isoquercetin. *International Journal of Applied Research in Natural Products*.

[191] Pitchai D., Manikkam R. (2012). Hypoglycemic and insulin mimetic impact of catechin isolated from *Cassia fistula*: a substantiate in silico approach through docking analysis. *Medicinal Chemistry Research*.

[192] Nazir N., Zahoor M., Ullah R., Ezzeldin E., Mostafa G. A. E. (2021). Curative effect of catechin isolated from *Elaeagnus umbellata* Thunb. Berries for diabetes and related complications in streptozotocin-induced diabetic rats model. *Molecules*.

[193] Ahangarpour A., Alboghobeish S., Oroojan A. A., Dehghani M. A. (2021). Caffeic acid protects mice pancreatic islets from oxidative stress induced by multi-walled carbon nanotubes (MWCNTs). *Veterinary Research Forum*.

[194] Huang D.-W., Shen S.-C., Wu J. S.-B. (2009). Effects of caffeic acid and cinnamic acid on glucose uptake in insulin-resistant mouse hepatocytes. *Journal of Agricultural and Food Chemistry*.

[195] Hsu F.-L., Chen Y.-C., Cheng J.-T. (2000). Caffeic acid as active principle from the fruit of Xanthium strumarium to lower plasma glucose in diabetic rats. *Planta Medica*.

[196] Zuñiga L. Y., Aceves-de la Mora M. C. A.-d., González-Ortiz M., Ramos-Núñez J. L., Martínez-Abundis E. (2018). Effect of chlorogenic acid administration on glycemic control, insulin secretion, and insulin sensitivity in patients with impaired glucose tolerance. *Journal of Medicinal Food*.

[197] Roshan H., Nikpayam O., Sedaghat M., Sohrab G. (2018). Effects of green coffee extract supplementation on anthropometric indices, glycaemic control, blood pressure, lipid profile, insulin resistance and appetite in patients with the metabolic syndrome: a randomised clinical trial. *British Journal of Nutrition*.

[198] Yan Y., Zhao X., Guo K., Zhou F., Yang H. (2020). Use of chlorogenic acid against diabetes mellitus and its complications. *Journal of Immunology Research*.

[199] Pérez-Nájera V. C., Gutiérrez-Uribe J. A., Antunes-Ricardo M. (2018). *Smilax aristolochiifolia* root extract and its compounds chlorogenic acid and astilbin inhibit the activity of *α*-amylase and *α*-glucosidase enzymes. *Evidence-Based Complementary and Alternative Medicine*.

[200] Oboh G., Agunloye O. M, Adefegha S. A, Akinyemi A. J, Ademiluyi A. O (2015). Caffeic and chlorogenic acids inhibit key enzymes linked to type 2 diabetes (*in vitro*): a comparative study. *Journal of Basic and Clinical Physiology and Pharmacology*.

[201] Raimundo A. F., Félix F., Andrade R. (2020). Combined effect of interventions with pure or enriched mixtures of (poly)phenols and anti-diabetic medication in type 2 diabetes management: a meta-analysis of randomized controlled human trials. *European Journal of Nutrition*.

[202] Pereira D. M., Valentão P., Pereira J. A., Andrade P. B. (2009). Phenolics: from chemistry to biology. *Molecular Diversity Preservation International*.

[203] Fakhruddin S., Alanazi W., Jackson K. E. (2017). Diabetes-induced reactive oxygen species: mechanism of their generation and role in renal injury. *Journal of Diabetes Research*.

[204] Ide T., Ashakumary L., Takahashi Y., Kushiro M., Fukuda N., Sugano M. (2001). Sesamin, a sesame lignan, decreases fatty acid synthesis in rat liver accompanying the down-regulation of sterol regulatory element binding protein-1. *Biochimica et Biophysica Acta (BBA) - Molecular and Cell Biology of Lipids*.

[205] Murase T., Mizuno T., Omachi T. (2001). Dietary diacylglycerol suppresses high fat and high sucrose diet-induced body fat accumulation in C57BL/6J mice. *Journal of Lipid Research*.

[206] Blakely S., Herbert A., Collins M. (2003). Lutein interacts with ascorbic acid more frequently than with *α*-tocopherol to alter biomarkers of oxidative stress in female zucker obese rats. *The Journal of Nutrition*.

[207] Ghosh D., Konishi T. (2007). Anthocyanins and anthocyanin-rich extracts: role in diabetes and eye function. *Asia Pacific Journal of Clinical Nutrition*.

[208] Ali Asgar M. (2013). Anti-diabetic potential of phenolic compounds: a review. *International Journal of Food Properties*.

[209] Mishio T., Takeda K., Iwashina T. (2015). Anthocyanins and other flavonoids as flower pigments from eleven Centaurea species. *Natural Product Communications*.

[210] Sulyok G., László-Bencsik Á. (1985). Cyanidin 3-(6-succinyl glucoside)-5-glucoside from flowers of seven Centaurea species. *Phytochemistry*.

[211] Kamanzi K. (1977). Les pigments anthocyaniques des fleurs de centaurea montana et de *Centaurea lugdunensis* (composees). *Plantes Medicinales et Phytotherapie*.

[212] Ames B. N., Shigenaga M. K., Hagen T. M. (1993). Oxidants, antioxidants, and the degenerative diseases of aging. *Proceedings of the National Academy of Sciences*.

[213] Levy Y., Glovinsky Y. (1998). The effect of anthocyanosides on night vision. *Eye*.

[214] Peterson J., Dwyer J. (1998). Flavonoids: dietary occurrence and biochemical activity. *Nutrition Research*.

[215] Hollman P. C. H., Katan M. B. (1999). Dietary flavonoids: intake, health effects and bioavailability. *Food and Chemical Toxicology*.

[216] Duthie G. G., Duthie S. J., Kyle J. A. M. (2000). Plant polyphenols in cancer and heart disease: implications as nutritional antioxidants. *Nutrition Research Reviews*.

[217] Cohen-Boulakia F., Valensi P. E., Boulahdour H. (2000). In vivo sequential study of skeletal muscle capillary permeability in diabetic rats: effect of anthocyanosides. *Metabolism*.

[218] Morimitsu Y., Kubota K., Tashiro T., Hashizume E., Kamiya T., Osawa T. (2002). Inhibitory effect of anthocyanins and colored rice on diabetic cataract formation in the rat lenses. *International Congress Series*.

[219] Galvano F., La Fauci L., Lazzarino G. (2004). Cyanidins: metabolism and biological properties. *The Journal of Nutritional Biochemistry*.

[220] Al-Awwadi N. A., Araiz C., Bornet A. (2005). Extracts enriched in different polyphenolic families normalize increased cardiac NADPH oxidase expression while having differential effects on insulin resistance, hypertension, and cardiac hypertrophy in high-fructose-fed rats. *Journal of Agricultural and Food Chemistry*.

[221] Jayaprakasam B., Vareed S. K., Olson L. K., Nair M. G. (2005). Insulin secretion by bioactive anthocyanins and anthocyanidins present in fruits. *Journal of Agricultural and Food Chemistry*.

[222] Ghosh D., McGhie T. K., Zhang J., Adaim A., Skinner M. (2006). Effects of anthocyanins and other phenolics of boysenberry and blackcurrant as inhibitors of oxidative stress and damage to cellular DNA in SH-SY5Y and HL-60 cells. *Journal of the Science of Food and Agriculture*.

[223] Varma S. D., Kinoshita J. H. (1976). Inhibition of lens aldose reductase by flavonoids—their possible role in the prevention of diabetic cataracts. *Biochemical Pharmacology*.

[224] Hammoud L., Seghiri R., Benayache S. (2012). A new flavonoid and other constituents from *Centaurea nicaeensis* all. var. Walliana M. *Natural Product Research*.

[225] Piesche M., Roos J., Kühn B. (2020). The emerging therapeutic potential of nitro fatty acids and other Michael acceptor-containing drugs for the treatment of inflammation and cancer. *Frontiers in Pharmacology*.

[226] Johnston G. (2009). Herbal products and GABA receptors. *Enclyclopedia of Neuroscience*.

[227] Dzoyem J. P., McGaw L. J., Bakowsky U. (2017). Anti-inflammatory and anti-nociceptive activities of african medicinal spices and vegetables. *Medicinal Spices and Vegetables from Africa*.

[228] Sakulnarmrat K., Konczak I. (2012). Composition of native Australian herbs polyphenolic-rich fractions and *in vitro* inhibitory activities against key enzymes relevant to metabolic syndrome. *Food Chemistry*.

[229] Khanavi M., Rajabi A, Behzad M, Hadjiakhoondi A, Vatandoost H, Abaee M. R (2011). Larvicidal activity of *Centaurea bruguierana* ssp. belangerana against *Anopheles stephensi* Larvae. *Iranian Journal of Pharmaceutical Research: IJPR*.

[230] Khanavi M., Ahmadi R., Rajabi A. (2012). Pharmacological and histological effects of *Centaurea bruguierana* ssp. belangerana on indomethacin-induced peptic ulcer in rats. *Journal of Natural Medicines*.

[231] Bashary R., Vyas M., Nayak S. K. (2020). An insight of alpha-amylase inhibitors as a valuable tool in the management of type 2 diabetes mellitus. *Current Diabetes Reviews*.

[232] Usman B., Sharma N., Satija S. (2019). Recent developments in alpha-glucosidase inhibitors for management of type-2 diabetes: an update. *Current Pharmaceutical Design*.

[233] Saeedi M., Hadjiakhondi A, Nabavi S. M, Manayi A (2017). Heterocyclic compounds: effective *α*-amylase and *α*-glucosidase inhibitors. *Current Topics in Medicinal Chemistry*.

[234] Shareghi-Boroujeni D., Iraji A., Mojtabavi S., Faramarzi M. A., Akbarzadeh T., Saeedi M. (2021). Synthesis, *in vitro* evaluation, and molecular docking studies of novel hydrazineylideneindolinone linked to phenoxymethyl-1,2,3-triazole derivatives as potential *α*-glucosidase inhibitors. *Bioorganic Chemistry*.

[235] Saeedi M., Raeisi-Nafchi M., Sobhani S. (2020). Synthesis of 4-alkylaminoimidazo [1, 2-a] pyridines linked to carbamate moiety as potent *α*-glucosidase inhibitors. *Molecular Diversity*.

[236] Saeedi M., Mohammadi-Khanaposhtani M., Asgari M. S. (2019). Design, synthesis, *in vitro*, and in silico studies of novel diarylimidazole-1,2,3-triazole hybrids as potent *α*-glucosidase inhibitors. *Bioorganic & Medicinal Chemistry*.

[237] Saeedi M., Mohammadi-Khanaposhtani M., Pourrabia P. (2019). Design and synthesis of novel quinazolinone-1,2,3-triazole hybrids as new anti-diabetic agents: *in vitroα*-glucosidase inhibition, kinetic, and docking study. *Bioorganic Chemistry*.

[238] Tundis R., Loizzo M. R., Menichini F. (2010). Natural products as *α*-amylase and *α*-glucosidase inhibitors and their hypoglycaemic potential in the treatment of diabetes: an update. *Mini-Reviews in Medicinal Chemistry*.

[239] Zhu J., Chen C., Zhang B., Huang Q. (2020). The inhibitory effects of flavonoids on *α*-amylase and *α*-glucosidase. *Critical Reviews in Food Science and Nutrition*.

[240] Bishoff H. (1985). Pharmacological properties of the novel glucosidase inhibitors BAY m 1099 (miglitol) and BAY o 1248. *Diabetes Research and Clinical Practice*.

[241] Apostolidis E., Kwon Y. I, Shetty K (2006). Potential of cranberry-based herbal synergies for diabetes and hypertension management. *Asia Pacific Journal of Clinical Nutrition*.

[242] Horii S., Fukase H., Matsuo T., Kameda Y., Asano N., Matsui K. (1986). Synthesis and *α*-D-glucosidase inhibitory activity of N-substituted valiolamine derivatives as potential oral antidiabetic agents. *Journal of Medicinal Chemistry*.

